# Multiple Origins and Regional Dispersal of Resistant *dhps* in African *Plasmodium falciparum* Malaria

**DOI:** 10.1371/journal.pmed.1000055

**Published:** 2009-04-14

**Authors:** Richard J. Pearce, Hirva Pota, Marie-Solange B. Evehe, El-Hadj Bâ, Ghyslain Mombo-Ngoma, Allen L. Malisa, Rosalynn Ord, Walter Inojosa, Alexandre Matondo, Diadier A. Diallo, Wilfred Mbacham, Ingrid V. van den Broek, Todd D. Swarthout, Asefaw Getachew, Seyoum Dejene, Martin P. Grobusch, Fanta Njie, Samuel Dunyo, Margaret Kweku, Seth Owusu-Agyei, Daniel Chandramohan, Maryline Bonnet, Jean-Paul Guthmann, Sian Clarke, Karen I. Barnes, Elizabeth Streat, Stark T. Katokele, Petrina Uusiku, Chris O. Agboghoroma, Olufunmilayo Y. Elegba, Badara Cissé, Ishraga E. A-Elbasit, Hayder A. Giha, S. Patrick Kachur, Caroline Lynch, John B. Rwakimari, Pascalina Chanda, Moonga Hawela, Brian Sharp, Inbarani Naidoo, Cally Roper

**Affiliations:** 1 London School of Hygiene & Tropical Medicine, Department of Infectious Tropical Diseases, London, United Kingdom; 2 Biotechnology Centre, University of Yaounde I, Cameroon; 3 Institut de Recherche pour le Développement, Dakar, Senegal; 4 Medical Research Unit, Albert Schweitzer Hospital, Lambaréné, Gabon; 5 Department of Parasitology, Institute of Tropical Medicine, University of Tübingen, Tübingen, Germany; 6 Ifakara Health Research and Development Center (IHRDC), Ifakara, Kilombero District, Tanzania; 7 Sokoine University of Agriculture, Department of Biological Sciences, Faculty of Science, Morogoro, Tanzania; 8 Doctors with Africa CUAMM Angola, Luanda, Angola; 9 Director of Uige Provincial Hospital, Uige Angola; 10 Centre National de Recherche et de Formation Sur Le Paludisme (CNRFP), Ouagadougou, Burkina Faso; 11 Médecins Sans Frontières, Manson Unit, London, United Kingdom; 12 Head of Malaria Department, Tigray Bureau of Health, Tigray, Ethiopia; 13 Médecins Sans Frontières – Ethiopia, Addis Ababa, Ethiopia; 14 Infectious Diseases Unit Division of Clinical Microbiology and Infectious Diseases, National Health Laboratory Service and School of Pathology, Faculty of Health Sciences, University of the Witwatersrand, Johannesburg, South Africa; 15 Medical Research Council Laboratories, The Gambia; 16 Kintampo Health Research Centre, Ghana; 17 Epicentre, Paris, France; 18 Institut de Veille Sanitaire, Paris, France; 19 Division of Clinical Pharmacology, Department of Medicine, University of Cape Town, Cape Town, South Africa; 20 Ministry of Health, Matola, Maputo Province, Mozambique; 21 National Malaria Control Programme, Ministry of Health and Social Services, Old State Hospital Grounds, Windhoek, Namibia; 22 National Hospital Abuja, Garki Abuja, Nigeria; 23 Université Cheikh Anta Diop de Dakar, Dakar, Senegal; 24 Malaria Research Centre, Department of Biochemistry, University of Khartoum, Khartoum, Sudan; 25 Department of Biochemistry, Faculty of Medicine and Medical Sciences, Arabian Gulf University, Manama, Kingdom of Bahrain; 26 Malaria Branch, Division of Parasitic Diseases, National Center for Zoonotic, Vector-Borne and Enteric Diseases, Centers for Disease Control and Prevention (CDC), Atlanta, Georgia, United States of America; 27 Ministry of Health of Uganda, National Malaria Control Programme, Kampala, Uganda; 28 National Malaria Control Centre, Lusaka, Zambia; 29 Malaria Research Lead Programme, Medical Research Council, Durban, South Africa; Joint Malaria Project, United Republic of Tanzania

## Abstract

**Background:**

Although the molecular basis of resistance to a number of common antimalarial drugs is well known, a geographic description of the emergence and dispersal of resistance mutations across Africa has not been attempted. To that end we have characterised the evolutionary origins of antifolate resistance mutations in the dihydropteroate synthase (*dhps*) gene and mapped their contemporary distribution.

**Methods and Findings:**

We used microsatellite polymorphism flanking the *dhps* gene to determine which resistance alleles shared common ancestry and found five major lineages each of which had a unique geographical distribution. The extent to which allelic lineages were shared among 20 African *Plasmodium falciparum* populations revealed five major geographical groupings. Resistance lineages were common to all sites within these regions. The most marked differentiation was between east and west African *P. falciparum*, in which resistance alleles were not only of different ancestry but also carried different resistance mutations.

**Conclusions:**

Resistant *dhps* has emerged independently in multiple sites in Africa during the past 10–20 years. Our data show the molecular basis of resistance differs between east and west Africa, which is likely to translate into differing antifolate sensitivity. We have also demonstrated that the dispersal patterns of resistance lineages give unique insights into recent parasite migration patterns.

## Abstract 2

Cally Roper and colleagues analyze the distribution of sulfadoxine resistance mutations and flanking microsatellite loci to trace the emergence and dispersal of drug-resistant *Plasmodium falciparum* malaria in Africa.

## Editors' Summary

**Background:**

*Plasmodium falciparum*, a mosquito-borne parasite that causes malaria, kills nearly one million people every year, mostly in sub-Saharan Africa. People become infected with *P. falciparum* when they are bitten by a mosquito that has acquired the parasite in a blood meal taken from an infected person. *P. falciparum* malaria, which is characterized by recurring fevers and chills, anemia (loss of red blood cells), and damage to vital organs, can be fatal within hours of symptom onset if untreated. Until recently, treatment in Africa relied on chloroquine and sulfadoxine–pyrimethamine. Unfortunately, parasites resistant to both these antimalarial drugs is now widespread. Consequently, the World Health Organization currently recommends artemisinin combination therapy for the treatment of *P. falciparum* malaria in Africa and other places where drug-resistant malaria is common. In this therapy, artemisinin derivatives (new fast-acting antimalarial agents) are used in combination with another antimalarial to reduce the chances of *P. falciparum* becoming resistant to either drug.

**Why Was This Study Done?:**

*P. falciparum* becomes resistant to antimalarial drugs by acquiring “resistance mutations,” genetic changes that prevent these drugs from killing the parasite. A mutation in the gene encoding a protein called the chloroquine resistance transporter causes resistance to chloroquine, a specific group of mutations in the dihydrofolate reductase gene causes resistance to pyrimethamine, and several mutations in *dhps*, the gene that encodes dihydropteroate synthase, are associated with resistance to sulfadoxine. Scientists have discovered that the mutations causing chloroquine and pyrimethamine resistance originated in Asia and spread into Africa (probably multiple times) in the late 1970s and mid-1980s, respectively. These Asian-derived mutations are now common throughout Africa and, consequently, it is not possible to determine how they spread across the continent. Information of this sort would, however, help experts design effective measures to control the spread of drug-resistant *P. falciparum*. Because the mutations in *dhps* that cause sulfadoxine resistance only began to emerge in the mid-1990s, they haven't spread evenly across Africa yet. In this study, therefore, the researchers use genetic methods to characterize the geographical origins and contemporary distribution of *dhps* resistance mutations in Africa.

**What Did the Researchers Do and Find?:**

The researchers analyzed *dhps* mutations in *P. falciparum* DNA from blood samples collected from patients with malaria in various African countries and searched the scientific literature for other similar studies. Together, these data show that five major variant *dhps* sequences (three of which contain mutations that confer various degrees of resistance to sulphadoxine in laboratory tests) are currently present in Africa, each with a unique geographical distribution. In particular, the data show that *P. falciparum* parasites in east and west Africa carry different resistance mutations. Next, the researchers looked for microsatellite variants in the DNA flanking the *dhps* gene. Microsatellites are DNA regions that contain short, repeated sequences of nucleotides. Because the number of repeats can vary and because microsatellites are inherited together with nearby genes, the ancestry of various resistance mutations can be worked out by examining the microsatellites flanking different mutant *dhps* genes. This analysis revealed five regional clusters in which the same resistance lineage was present at all the sites examined within the region and also showed that the resistance mutations in east and west Africa have a different ancestry.

**What Do These Findings Mean?:**

These findings show that sulfadoxine-resistant *P. falciparum* has recently emerged independently at multiple sites in Africa and that the molecular basis for sulfadoxine resistance is different in east and west Africa. This latter result may have clinical implications because it suggests that the effectiveness of sulfadoxine as an antimalarial drug may vary across the continent. Finally, although many more samples need to be analyzed to build a complete picture of the spread of antimalarial resistance across Africa, these findings suggest that economic and transport infrastructures may have played a role in governing recent parasite dispersal across this continent by affecting human migration. Thus, coordinated malaria control campaigns across socioeconomically linked areas in Africa may reduce the African malaria burden more effectively than campaigns that are confined to national territories.

**Additional Information:**

Please access these Web sites via the online version of this summary at http://dx.doi.org/10.1371/journal.pmed.1000055.

- This study is further discussed in a *PLoS Medicine* Perspective by Tim Anderson
- The MedlinePlus encyclopedia contains a page on malaria (in English and Spanish)
- Information is available from the World Health Organization on malaria (in several languages) and on drug-resistant malaria
- The US Centers for Disease Control and Prevention provide information on malaria (in English and Spanish)
- Information is available from the Roll Back Malaria Partnership on its approach to the global control of malaria, and on malaria control efforts in specific parts of the world
- The WorldWide Antimalarial Resistance Network is creating an international database about antimalarial drug resistance

## Introduction

Chloroquine (CQ) and the antifolate combination of sulphadoxine–pyrimethamine (SP) were, until recently, the mainstay of malaria treatment in Africa. Resistance to both drugs is now widespread. In both cases the importation of resistance mutations to Africa from Asia played a decisive role in the establishment of resistance [1]–[3], but details of where, when, or how resistance genes were introduced in Africa are unknown.

CQ was first used in the 1950s, and chloroquine resistance (CQR) appeared in Asian and south American foci in the early 1960s. CQR did not appear in Africa until 1978, when the initial focus was in east Africa. It subsequently appeared to radiate from that focus, reaching west Africa between 1986 and 1989 [4], [5]. The major genetic determinant of CQR is now known to be the *P. falciparum* chloroquine resistance transporter (encoded by the *pfcrt* gene) [6], and microsatellites in the flanking sequence around *pfcrt* resistance alleles show that the Asian lineage of mutant *pfcrt* is present in *P. falciparum* populations throughout Africa [3], [7]. It is probable that resistant *pfcrt* was introduced to Africa on multiple occasions, as CQR was common throughout Asia by that time [4], but archived samples from 30 years ago are rare, making a retrospective analysis of the geographical dispersal of resistant *pfcrt* in Africa intractable.

SP began to be used for treatment of CQR malaria in Africa in the 1980s. Resistance to SP involves adaptations in the target molecules, dihydrofolate reductase (DHFR) (the target of pyrimethamine) [8], [9] and dihydropteroate synthase (DHPS) (the target of sulphadoxine) [10], [11]. The triple-mutant *dhfr* (containing mutations N51I+C59R+S108N) confers a significant component of resistance to SP [12]. It is found throughout Africa [13]–[18] and is derived from a single ancestor, which originated in Southeast Asia [1]. Analysis of archived parasite specimens show that the triple-mutant lineage was present at least as early as 1985 in Cameroon [16] and at least as early as 1988 in Kenya [18]. Like *pfcrt*, resistant *dhfr* may have been imported on multiple occasions, but historical samples are limiting and since the Asian-type sequence is now common throughout Africa it is not possible to ascertain any geographical detail about its arrival and dispersal on the continent more than 20 years ago.

Although the pan-African distribution of Asian-derived *pfcrt* and *dhfr* lineages today suggests that the *P. falciparum* populations of Africa form one large continuous whole, the geography of their introduction 20–30 years ago and their subsequent dispersal has not been characterised. The emergence of *dhps* resistance alleles has occurred more recently than either *pfcrt* or *dhfr*, creating an opportunity to directly observe resistance dispersal events while they are in progress.

Clinical treatment failure with SP was first reported in Tanzania in 1995 [19], [20], and the timing of its emergence in Africa broadly coincides with the first appearance of mutant *dhps* against a pre-established background of resistant *dhfr*. In east Africa, mutations at codons A437G and K540E of *dhps*, together with the triple mutations of *dhfr* were shown to be a significant predictor of SP treatment failure in Kenya, Malawi, and Uganda [21]–[23]. In west Africa, where the K540E mutation is rare, an association of treatment failure with the A437G plus the triple-mutant *dhfr* has been reported by studies in Gabon [24], Ghana [25], The Gambia [26] and Congo (Brazzaville) [27]. In contrast, a study in Ghana by Marks et al. reported no association of A437G with treatment outcome [28].

**Figure 1:**
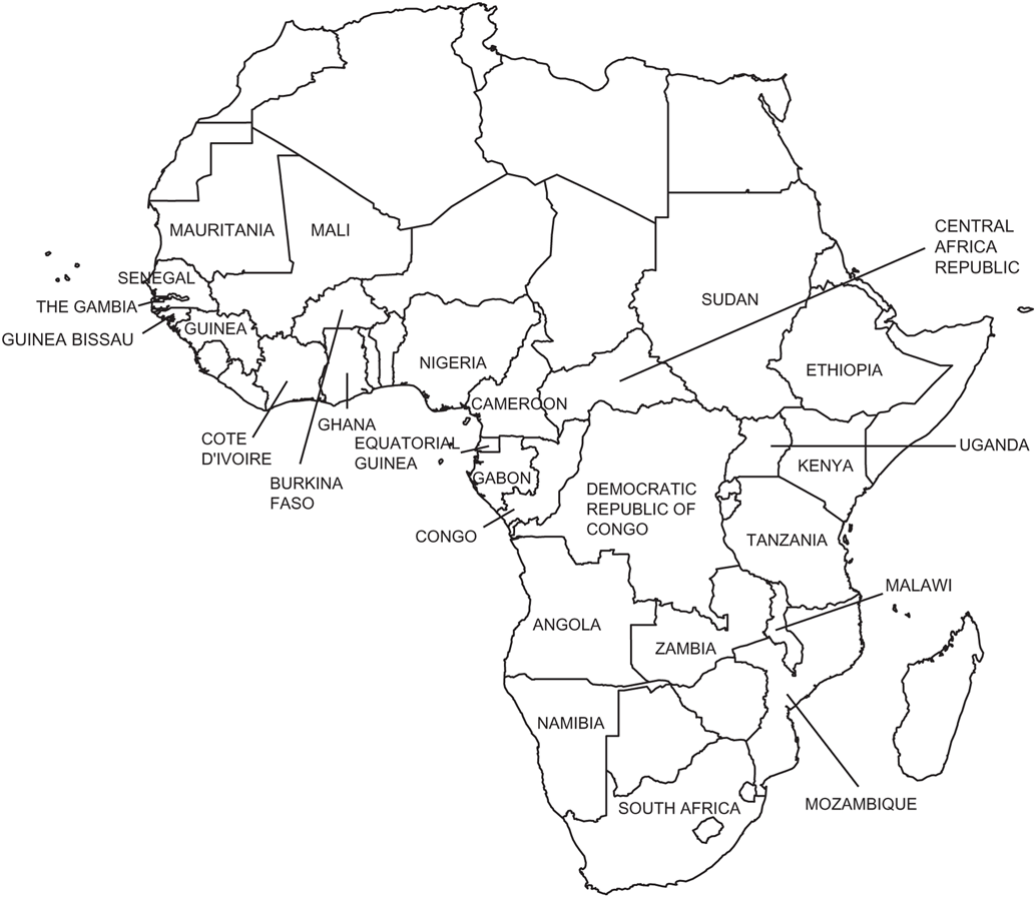
Map of the countries of Africa included in this study.

The spatial distribution of *dhps* mutations in Africa has not previously been mapped. To obtain the highest resolution possible, we generated new SNP data for 20 countries and combined this with additional data from seven published studies to cover a total of 50 sites in the 27 countries shown in Figure 1. To investigate the evolutionary origins of *dhps* mutations we examined diversity at microsatellite markers flanking the gene and used this to characterise lineages of common ancestry that have been subject to recent selection. In this way we were able to describe the dispersal dynamics of resistance alleles currently under selection and to generate new hypotheses about the geography of malaria migration in modern Africa.

## Materials and Methods

### Study Sites

Study sites are listed in Table 1. The details of sample collection procedures and ethical permissions at every site for which new data are described here are detailed in Text S1. Literature searches were done during June 2007 and updated in October 2007 using the National Library of Medicine search engines, Pub Med and Medline (details given in Text S2). We identified 20 published studies in which *dhps* point mutation haplotypes including codons 436, 437, and 540 had been reported in *P. falciparum* isolates sampled in Africa since 1997 (Table 1).

**Table 1:** Study site details and numbers of data points included.

Country	Study Site	Samples Successfully Typed at *dhps*, *n*	Samples Successfully Typed at Microsatellite Loci and *dhps*, *n*	Reference^a^
**Angola**	Uige Province	40	39	This study
**Burkina Faso**	Bousse	365	100	This study
	Nanoro	60	—	[60]
**Cameroon**	Garoua	71	—	This study
	Yaounde	143	98	This study
	Mutengene	202	183	This study
**Central African Republic**	Bangui	74	—	[61]
**Congo**	Pointe Noire and Brazzaville	135	—	[62]
	Kindamba	236	154	This study and [63]
**Cote d'Ivoire**	Yopougon Abidjan	118	—	[35]
**DRC**	Shabunda	117	67	This study and [64]
**Equatorial Guinea**		12	—	[65]
**Ethiopia**	Dilla	69	—	[39]
	Humera	87	38	This study
	Jimma	124	—	[38]
**Gabon**	Haut-Ogooue	82	—	[66]
	Lambarene	64	62	This study
**Gambia**	Farafenni	127	—	This study
**Ghana**	Navrongo	101	95	This study
	Hoehoe	126	—	This study
**Guinea**	Laine	114	56	This study and [67]
**Guinea Bissau**	Bandim	91	—	[68]
**Kenya**	Bondo	133	111	This study
**Malawi**	Salima	159	—	[69]
**Mali**	All sites	13	—	[70]
**Mauritania**	Aioun and Kobeni	160	—	[71]
**Mozambique**	East Rural	110	110	This study
	Periurban	134		This study
	West Rural	96		This study
**Namibia**	Kavango	76	75	This study
**Nigeria**	Abuja	17	15	This study
**Senegal**	Pikine	15	—	[14]
	Niakar	234	44	This study
**South Africa**	Ingwavuma	198	27	[13]
	Komatipoort	306	—	This study
**Sudan**	Lankien	44	—	[72]
	Yargot Payam Bahr el Gazal	75	—	[54]
	Gedaref	69	68	This study
**Tanzania**	Hai	81	—	[29]
	North Pare	30	—	[29]
	South Pare	33	—	[29]
	Kilombero and Ulanga	561	89	This study and Malisa et al., personal communication
**Uganda**	Kabale and Rukungiri	129	129	This study and [17]
**Zambia**	Chibombo	15	114	This study
	Chipata	12		This study
	Chongwe	58		This study
	Isoka	54		This study
	Mansa	22		This study
	Mpongwe	24		This study

a Where more than one reference was available per country the most recent was taken.

### SNP Genotyping

In all studies finger-prick blood spots were taken from infected individuals and parasite DNA was extracted from the blood spots using the Chelex method, and the polymorphic region of *dhps* was PCR-amplified prior to sequence-specific oligonucleotide probing (SSOP) for polymorphism at codons 436, 437, and 540. The DNA extraction, PCR amplification, and SSOP dot-blotting procedures have been described previously [29]. Since blood-stage *P. falciparum* is haploid, the determination of allelic haplotypes is straightforward when an infection consists of a single genotype, because only one form of sequence at every SNP is seen. When infections are composed of multiple genotypes, however, the mixture of different sequence variants makes inference of point mutation haplotypes within that infection more difficult. A sample was considered to have a single haplotype when only one sequence variant was found at each locus. In mixed-genotype infections, if one genotype was substantially in the majority (i.e., the hybridisation signal of the minority sequence was less than half the intensity of the majority), then the majority haplotype was recorded. One haplotype only was counted from each infection, and those mixed infections for which haplotypes could not be resolved were omitted from the calculation of haplotype frequencies (numbers of mixed infections excluded from each sample are given in Table S1). It should be noted that the rate of detection of mixtures is dependent on the extent of polymorphism at a given locus. Furthermore, sampling at various study sites was not standardised for factors known to affect rates of mixture, namely, patient age, rates of self treatment before attendance at the health facility, and transmission intensity itself.

### Maps of Africa

Maps of the distribution of *dhps* alleles were constructed using MapInfo (MapInfo Limited, Windsor, United Kingdom).

### Microsatellite Analysis

Microsatellite loci flanking *dhps* at 0.8 kb, 4.3 kb, and 7.7 kb from the 3′ end of the gene were amplified in samples from 20 sites across 19 countries. Full primer sequences and cycling conditions can be found in [13]. The amplification products were run diluted 1∶100 and run with LIZ-500 size standard on the ABi 3730 DNA analyser (Applied Biosystems, Foster City, California, United States) and analysed using the software Genemapper (Applied Biosystems). In samples where more than one allele was present at a locus the data was considered missing at that locus for that sample.

### Heterozygosity Analysis

Gene diversity values were calculated as 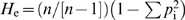, where *H*_e_ is expected heterozygosity, *n* is the number of samples, and *p*_i_ is the frequency of the *i*th allele in the sample set. Heterozygosity of microsatellites flanking each of the five key *dhps* alleles was calculated for each separate study site. Geographic sites at which the sample size was less than ten were not included. The box plots were constructed in the statistical package R [30], which calculates the median and interquartile intervals. The total range of the distribution of heterozygosity values was described by the upper and lower extreme values, provided they fell within a range that was calculated as 1.5× the interquartile range below the first and above the third quartiles. Extreme values that fell outside this distribution were identified as outliers and plotted separately. The statistical significance of the loss of diversity around mutant *dhps* alleles was calculated by Wilcoxon's rank sum test which compared the variability of *H*e among geographical sites with equivalent *H*_e_ values for the same microsatellite flanking the wild-type allele (haplotype SAK at codons 436, 437, and 540).

### Population Comparison of Resistance Allele Sharing

To examine the extent to which resistance alleles were exchanged among the populations at 20 sites we used a pairwise population measure of resistance allele sharing. For the analysis we expressed the combination of point mutations and linked microsatellite alleles at the 0.8 kb and 4.3 kb loci as a single allelic haplotype, and estimated *D*_PS_ for a single locus. *D*_PS_ was calculated as 1−*ps* (where *ps* is the sum of the minima of the relative frequencies of all alleles shared between compared population samples) [31].
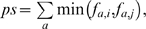
(1)where 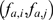 is the frequency of allele *a* in populations i and j.

Resistance allele sharing among the 20 geographical sites is summarised in the neighbour joining tree generated in the neighbour package of Phylip [32].

### Analysis of Diversity Flanking Wild-Type Alleles

To compare heterozygosity at the 0.8 kb microsatellite locus linked to wild-type alleles with haplotypes SAK and AAK at codons 436, 437, and 540, we used a method described by Nash et al. [33]. Significance was determined by comparing the ratio of heterozygosity (*H*_e_ SAK/*H*_e_ AAK) observed in each geographical region with the ratio of heterozygosities from 10,000 simulated datasets in which microsatellite alleles were reshuffled amongst all parasites. To obtain the level of significance for the difference in *H*_e_, the number of occasions that the simulated ratio of heterozygosities exceeded the observed was counted and converted to the proportion of the 10,000 simulated datasets.

For assessing the relationship between the sensitive chromosomes and the single-mutant AAK chromosomes, Nei's standard genetic distance [34] was calculated for all pairwise comparisons in Phylip [32]. The significance of the observed standard genetic distance between allelic populations was determined by comparison to genetic distance values from 10,000 simulated datasets in which the alleles at each locus were reshuffled among all parasites. To obtain the level of significance, the number of occasions that the simulated distance exceeded that for the observed data was counted, then converted to the proportion of the 10,000 simulated datasets. The statistical package R [30] was used to permutate the datasets.

## Results

### The Geographical Distribution of *dhps* Mutation Haplotypes

To map the distribution of *dhps* mutations in Africa we collected and typed *P. falciparum* DNA extracted from finger-prick samples from 3,761 malaria patients at 31 sites in 20 African countries. The details of individual studies are described in full in Text S1. Where possible these data were supplemented with previously published data from the literature (the complete list of survey sites is given in Table 1). We combined original and published data to obtain the point mutation haplotypes for codons 436, 437, and 540 of *dhps* for 5,493 unmixed isolates collected between 1997 and 2007 at 50 unique geographical locations in Africa. Blood stage parasites are haploid, so where infections consist of a single genotype it is possible to determine complete haplotype information. This determination is not possible with blood samples from patients with mixed-genotype infections. Among the pooled samples from all geographic locations together we found five major point mutation haplotypes; SGE, AGK, SGK, AAK, and SAK. In addition there were rare single-mutant haplotypes coding for alternative substitutions at position 436. We found 67 examples of the 436F mutation dispersed across 14 geographical populations, seven examples of 436C in five countries, and 13 isolates in Cote d'Ivoire with a 436Y substitution which were previously described by Djaman et al. [35].

**Figure 2:**
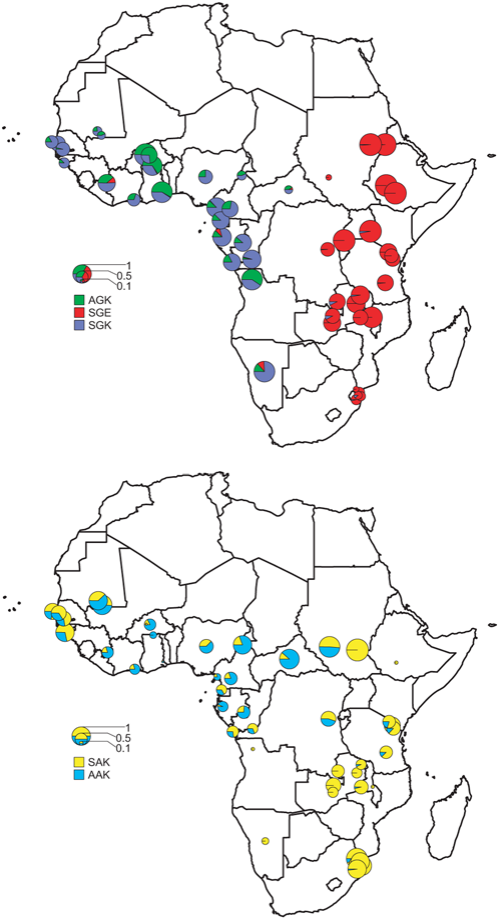
The distribution of the major *dhps* alleles across sub-Saharan Africa. Resistant alleles; the upper map shows the relative proportions of the three major resistance alleles, SGK, AGK, and SGE. Wild-type alleles; the lower map shows the ratio of SAK and AAK alleles among wild-type *dhps* alleles. In both cases the diameter of the pie is proportional to the combined frequencies of the alleles represented in the total population.

The SAK is generally regarded as the ancestral wild type, and alleles that contain the S436A alone (AAK) are considered alternative wild types [36]. All haplotypes containing the A437G substitution (SGE, AGK, SGK) are known to confer resistance to sulphadoxine in vitro [37], and on that basis we classified the five haplotypes as either wild type (SAK and AAK) or resistant (SGE, AGK, and SGK). Figure 2 shows the geographical distribution of resistant alleles SGK, AGK, and SGE and wild-type alleles AAK and SAK. A complete listing of the frequencies of all haplotypes at every site is given in Table S1.

The size of the pie charts in the upper map of Figure 2 indicates the proportion of the total sample that was resistant. Sites where resistance allele frequencies were high relative to wild-type alleles are indicated by large pie charts, while those where resistance alleles were less abundant than wild type are indicated by small pie charts. The abundance of resistance alleles varied from site to site presumably in accordance with local malaria drug treatment practices. Selection for resistance has clearly been high in both east and west Africa. Although there was no obvious geographical trend in the ratios of resistant to wild-type alleles, there was a very striking difference in the type of resistance alleles that were prevalent in east and west Africa, illustrated by the coloured segments of the pie charts in the upper map of Figure 2. In east Africa, the A437G and K540E substitutions occurred together as a double-mutant allele SGE (shown in red). In west Africa, the A437G substitution was found alone as either an SGK (blue) or an AGK (green) allele, and the SGE allele was rare or absent.

The SGE allele was prevalent in a number of east African sites, and its frequency exceeded 95% of the total parasite population in Kenya 2006 (this study), Uganda 2005 (this study), Sudan 2003 (this study), and three sites in Ethiopia 2004 (this study and [38], [39]). The SGE allele was rare (0%–9%) in central and southwest African samples (Gabon 2007, Cameroon 2004, Congo 2004, Central African Republic 2004, Angola 2004, and Namibia 2005) and absent in many west African samples (Nigeria 2005, Cote d'Ivoire 2001, Burkina Faso 2002, Burkina Faso 2003, The Gambia 2004, Mauritania 1998, Mali 1997, Senegal 2003, and Senegal 2004) although small numbers of SGE were detected in three west African samples: Guinea 2004–2005 (7%); Navrongo, Ghana 2003 (<1%); and Hoehoe, Ghana 2005 (<1%).

In west, central, and southwest Africa, instead of SGE, the SGK and AGK resistance alleles prevailed, often at high frequency; for example, in the samples from Nanoro, Burkina Faso 2003 (98%); Hohoe, Ghana 2005 (94%); Angola 2004 (92%); Navrongo, Ghana 2003 (85%); Mutengene, Cameroon 2004 (84%); Namibia 2005 (78%); Congo 2004 (72%); Gabon 2007 (69%); Guinea 2004–2005 (59%); and Bousse, Burkina Faso 2002 (57%). The combined frequency of SGK and AGK resistance alleles was intermediate or low in other samples such as Senegal 2004 (50%), Nigeria 2005 (47%), The Gambia 2004 (46%), Senegal 2003 (40%), Cote d'Ivoire 2001 (39%), Central African Republic 2004 (15%), Mauritania 1998 (18%), and Mali 1997 (0%).

The frequency of wild-type alleles in the total population inevitably reflects the history of recent antimalarial drug use at any given site, because where the drug selection has been intense, wild-type alleles are increasingly displaced by resistant alleles. In the lower map of Figure 2 the size of the pie chart at each site indicates the proportion of wild-type alleles in the total population as indicated in the scale. The largest pie charts indicate populations in which the majority of parasite isolates were found to be wild type, and the smallest pie charts indicate where the wild types were rare. The relative proportions of SAK and AAK alleles within the wild-type population are indicated by the yellow and cyan segments. The AAK allele was most common in central Africa, accounting for more than half of the total wild-type alleles in Gabon 2007; Burkina Faso 2002; Guinea 2004–2005; Ghana 2005; Nigeria 2005; Mutengene, Cameroon 2004; Yaounde, Cameroon 2004; and Central African Republic 2004. Moving out from this area, the relative proportion of wild-type alleles that were AAK decreased. In the west; 28% of wild-type alleles were AAK in The Gambia 2004, 22% in Senegal 2003, 34% in Senegal 2004, and 53% in Mauritania 1998. Moving south, the proportion of AAK becomes increasingly rare, accounting for just 13% of wild-type alleles in Tanzania,6% in South Africa, and 5% in Mozambique; and absent in Namibia (0%) and Angola (0%).

### Selective Sweeps around Resistant *dhps* Alleles

**Figure 3:**
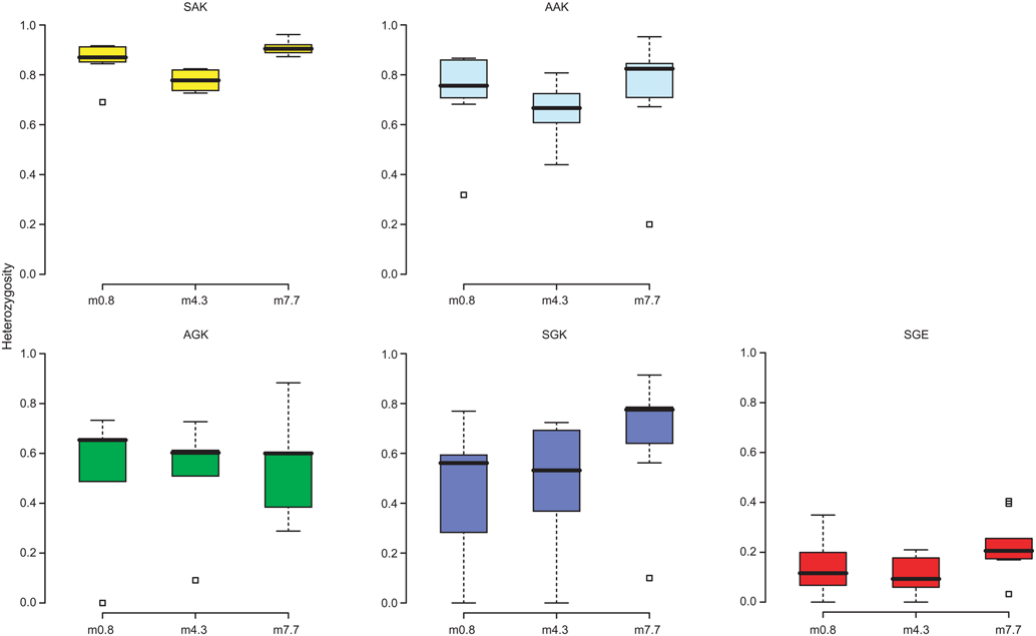
Microsatellite diversity around the wild-type (SAK and AAK) and the resistant (AGK, SGK, and SGE) alleles. The expected heterozygosity (*H*_e_) at flanking loci 0.8 kb, 4.3 kb, and 7.7 kb from the *dhps* gene was calculated for each geographical site (provided the number of observation ≥10), and box plots show the median, interquartile ranges, and the upper and lower extremes of the distribution of *H*_e_ values among geographical sites. Where there are statistical outliers, these are indicated by small squares.

To confirm that resistant alleles had been subject to selection and to classify alleles according to shared ancestry we examined microsatellite diversity in the flanking region of *dhps*. Three microsatellite loci at 0.8 kb, 4.3 kb, and 7.7 kb from the 3′ end of *dhps* were successfully analysed in 1,674 unmixed samples from 20 geographical sites (the raw data are listed in full in the Table S2). A loss of diversity around resistant alleles, compared to that found surrounding the wild-type alleles, is evidence of directional selection and often referred to as a selective sweep. The expected heterozygosity (*H*_e_) around each *dhps* allele at each geographical site was calculated extending outwards from the *dhps* gene and values are presented for loci at 0.8 kb, 4.3 kb, and 7.7 kb. In Figure 3 the diversity around wild-type (SAK and AAK) and resistant (AGK, SGK, and SGE) alleles are compared. The range of *H*_e_ values around the median are illustrated by box distributions, which show the interquartile range, and the whiskers, which show upper and lower extremes of the distribution.

Diversity around wild-type alleles was universally high with little variation among geographical sites. Also there was no significant change in diversity with distance along the chromosome from either the SAK or the AAK *dhps* alleles, which is consistent with expectations for a locus that has not been subject to recent selection. The median values for *H*_e_ around the SAK alleles were 0.867 (0.8 kb), 0.778 (4.3 kb), and 0.905 (7.7 kb). Similarly, the diversity around AAK was high: 0.756 (0.8 kb), 0.667 (4.3 kb), and 0.824 (7.7 kb).

Contrasting with wild-type *dhps* alleles there were clear signatures of selection around all three alleles coding for the A437G substitution, confirming that these alleles have been subject to selection. Loss of diversity was most pronounced at loci flanking the SGE double-mutant allele, where *H*_e_ values were 0.116 (0.8 kb), 0.093 (4.3 kb), and 0.206 (7.7 kb). These values are significantly different from the *H*_e_ flanking the SAK haplotype (*p*<0.001 at all three sites when compared using Wilcoxon's rank sum test). There was also a loss of diversity around the AGK alleles, where equivalent *H*_e_ values were 0.653 (*p* = 0.005), 0.603 (*p* = 0.003), and 0.600 (*p* = 0.01), respectively, and SGK alleles, where *H*_e_ values were 0.561 (*p* = 0.0007), 0.532 (*p* = 0.0002), and 0.775 (*p* = 0.0115), respectively. The variability of *H*_e_ values among geographical populations was more pronounced with AGK and SGK alleles than for SGE alleles; this difference may be due to multiple lineages occurring within individual populations. “Soft” selective sweeps are found where multiple lineages are superimposed within a single population [40], causing *H*_e_ to be higher than in populations where a single lineage is present. We went on to examine how many lineages could be identified and to examine their geographical distribution among populations.

### Multiple Origins of Resistant *dhps*

**Figure 4:**
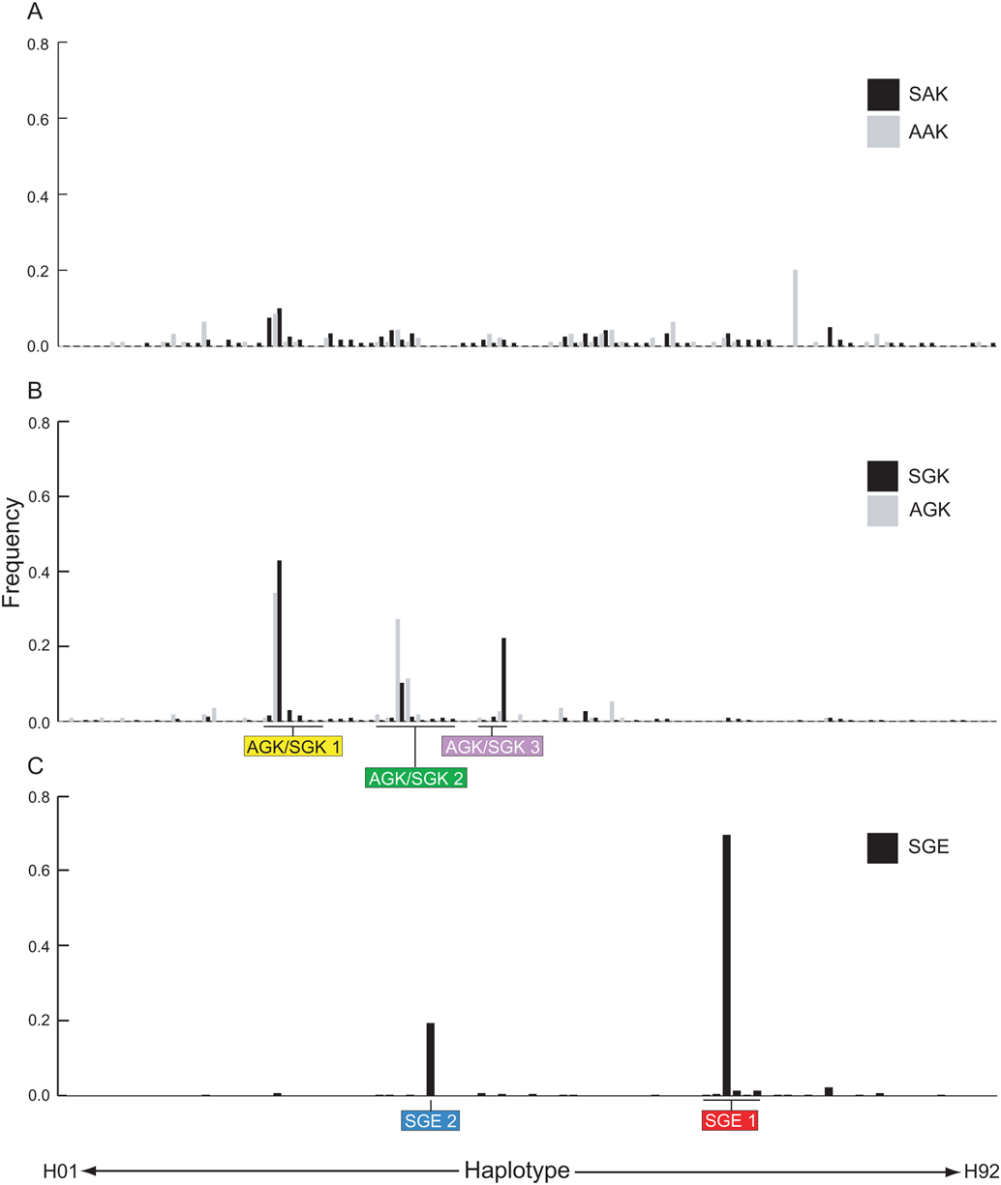
Microsatellite polymorphism flanking wild-type and resistant *dhps* alleles. In the bar graphs all the microsatellite haplotypes observed have been ranked first according to allele size at locus 0.8 kb and then by allele size at locus 4.3 kb along a common *x*-axis. The association of specific microsatellite haplotypes with different *dhps* alleles is apparent from the frequencies of each haplotype shown in the individual charts. (A) haplotypes linked to SAK AAK wild-type alleles, (B) haplotypes linked to AGK SGK single-mutant alleles, and (C) haplotypes linked to SGE double-mutant alleles.

Resistance mutations that have common ancestry can be identified on the basis of flanking microsatellite polymorphism, because closely linked neutral markers are carried with the selected allele by hitch-hiking. Haplotypes of the two most closely linked markers (0.8 kb and 4.3 kb) were ranked, first according to allele size at locus 0.8 kb and then by allele size at locus 4.3 kb. In Figure 4 the ranked microsatellite haplotypes are listed along a common *x*-axis in three bar charts, which show their frequency among sensitive (Figure 4A), single 437 mutant alleles SGK and AGK (Figure 4B), and double-mutant 437+540 SGE alleles (Figure 4C). A complete list of microsatellite haplotypes is available in the Table S2.

The microsatellite haplotypes associated with SAK and AAK alleles (Figure 4A) were largely unique to every isolate, which is consistent with the expectation that they have not been under recent selection. In contrast, many of the resistance alleles were found to share a common flanking microsatellite haplotype, indicating that they have been under recent selection and that they were derived from the same ancestral mutant lineage. Inspection of the microsatellite haplotypes associated with SGK and AGK alleles (Figure 4B) shows that multiple lineages have emerged, and within these the S436A mutation appears to have been gained or lost on multiple occasions. Three lineages of an AGK/SGK allele were identified, shown in Figure 4B as AGK/SGK 1, AGK/SGK 2, and AGK/SGK 3. These lineages were defined on the basis of a shared allele size at the most proximal (0.8 kb) microsatellite locus. Occasional recombination in the flanking region at sites more distant from the gene accounted for some variability at the 4.3 kb locus, and isolates contained within these clusters of related haplotypes are highlighted by underlining in Figure 4B.

Two major clusters of microsatellite haplotypes were associated with the SGE double-mutant allele (Figure 4C). Within each cluster the microsatellite haplotypes all share the same allele at the closest microsatellite locus (0.8 kb), but may vary at locus 4.3 kb because of the increasing likelihood of recombination events with distance from the site of selection. Lineages defined on the basis of variation at the 0.8 kb locus are highlighted by underlining and named SGE1 and SGE2 in Figure 4C.

### The Geographic Dispersal of Resistant Lineages

To examine the extent to which resistance alleles were exchanged among the populations at the 20 sites, we used pairwise population measure of resistance allele sharing. For the analysis we expressed the combination of point mutations and linked microsatellite alleles at the 0.8 kb and 4.3 kb loci as a single haplotype, and estimated *D*_PS_ for a single locus.

**Figure 5:**
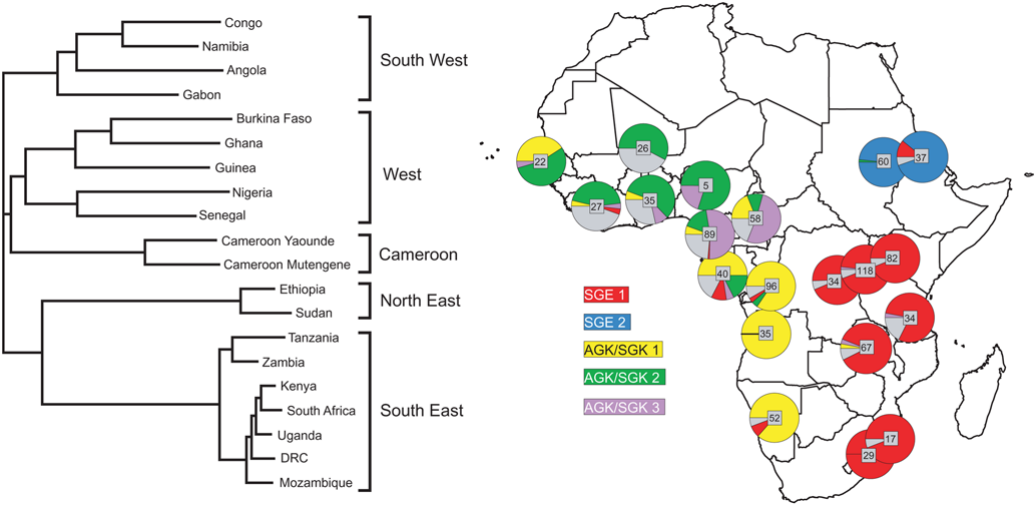
The African distribution of *dhps* resistance lineages. The distribution of the five major lineages among the geographic sites is indicated in the map. Resistance alleles whose flanking microsatellite haplotypes did not conform to a defined major lineage are shown in grey. Sharing of resistance allele lineages among the African populations is shown in a cladogram based on pairwise comparison of allele sharing (*D*_PS_), which includes all the flanking haplotypes identified. Closely related populations cluster in large geographic regions that supercede national boundaries.

Resistance allele sharing among the 20 geographical sites is summarised in the neighbour joining tree in Figure 5. Resistance allele sharing among the 20 sites reveals five regional clusters. Within these clusters the same lineages are common, but between them few or none of the resistance allele lineages were shared. The geographical distribution of resistance allele lineages and their representation in each of the 20 geographical sites are illustrated in the map in Figure 5. The geographical distribution of AGK/SGK 1, AGK/SGK 2, and AGK/SGK 3 lineages were each unique, with the concentration of each lineage implying their likely site of origin. The lineage AGK/SGK 1 was found predominantly in central and southwest African sites, Namibia, Angola, Congo, and Gabon, AGK/SGK 2 was found predominantly in the west African sites Senegal, Guinea, Burkina Faso, Ghana, and Nigeria, and AGK/SGK 3 was found predominantly in Cameroon. It is clear from the frequencies of resistance lineages expressed in the pie chart map in Figure 5 that there has been dispersal throughout west and central Africa from their original foci, with Cameroon at the confluence of west, central, and southwest African gene pools.

The flanking haplotypes associated with SGE alleles revealed the existence of two lineages SGE1 and SGE2. A previous study has shown that SGE mutants from South Africa and northern Tanzania were derived from a one ancestral lineage [13]. We found that the geographic range of that lineage (SGE1) not only includes South Africa and Tanzania but extends through Kenya, Uganda, Democratic Republic of Congo (DRC), Mozambique, and Zambia. Interestingly, the SGE alleles in Ethiopia and northeastern Sudan are descendants of an independently derived and regionally distinct lineage (SGE2).

### Microsatellite Diversity Flanking Wild-Type *dhps* Alleles

Polymorphism at codon 436 is generally considered to be the ancestral state, and the high levels of diversity measured at the microsatellite loci flanking the SAK and AAK alleles when all geographical populations were compared was consistent with the ancestral sensitive state. There was, however, some evidence of weak selection acting upon the AAK allele when parasites from individual geographical sites are examined separately. One flanking haplotype was found at relatively high frequency among the samples with the AAK allele at *dhps* (this lineage is defined as type H73 in Table S2) in Cameroon (Yaounde *n* = 9, Mutengene *n* = 3), Gabon (*n* = 5), and Nigeria (*n* = 2), indicating a recently selected expansion of this allele in that region. We infer that selection was attributable to the mutation at codon 436. We tested for additional mutations at codons 581 and 613 in these samples and none were found, but we cannot entirely exclude the possibility that other linked adaptations [41] might be involved.

If the 436A mutation consistently confers a selective advantage, we would predict that the reduction of diversity around AAK would be detectable in other geographic regions. To test this prediction we compared the diversity in the 0.8 kb microsatellite locus linked to the SAK and AAK alleles in each region. The northeast Africa region was excluded from this analysis because AAK and SAK alleles were very rare in the Ethiopia and Sudan samples. Table 2 shows a significant loss of heterozygosity at the 0.8 kb locus among AAK alleles relative to SAK alleles in all regions except the southeast. The margin of difference was greatest in Cameroon and in southwest Africa (*p*-values are shown in Table 2).

**Table 2:** Expected heterozygosity *H*_e_ at the 0.8 kb microsatellite locus linked to SAK and AAK single-mutant alleles by geographical region.

Region^a^	SAK	AAK	*p*-Value	% Reduction *H*_e_
Cameroon	0.844 (*n* = 10)	0.756 (*n* = 29)	*p*<0.0001	10.4%
Southeast Africa	0.909 (*n* = 101)	0.860 (*n* = 17)	*p* = 0.433	5.4%
Southwest Africa	0.757 (*n* = 31)	0.688 (*n* = 24)	*p*<0.0001	9.1%
West Africa	0.937 (*n* = 20)	0.882 (*n* = 50)	*p*<0.0001	5.9%

The significance of the difference in *H*_e_ between SAK and AAK alleles is shown for each region together with the percentage reduction in *H*_e_. The significance of the difference in diversity was determined by permutation and p values express the number of times the observed ratio of diversity between SAK and AAK was met or exceeded in 10,000 simulated datasets.

a Populations included in these regions are shown in Figure 5.

The significant reduction in diversity around AAK relative to SAK in each region suggests that multiple lineage expansion events have occurred independently in the different regions. If this were the case we would expect to see greater regional differentiation among the AAK-linked microsatellite haplotypes than among those linked to sensitive SAK alleles. Pairwise genetic identity among SAK-linked microsatellite haplotypes from the four regions was calculated using all three flanking microsatellites. Table 3 indicates that they are all of a similar level of identity, whereas pairwise comparisons of populations using genetic identity among AAK-linked microsatellites in the three populations have much lower identity (Table 3). The greater dissimilarity among AAK was a significant departure from expected (test by permutation).

**Table 3:** Regional differentiation at microsatellite variation linked to SAK and AAK alleles as calculated by Nei's standard genetic distance.

Region^a^	Cameroon		Southeast Africa		Southwest Africa	
	SAK	AAK	SAK	AAK	SAK	AAK
Southeast Africa	0.677 (*p* = 0.069)	0.291 (*p*<0.0001)	—	—	—	—
Southwest Africa	0.724 (*p* = 0.147)	0.563 (*p*<0.0001)	0.785 (*p* = 0.003)	0.451 (*p*<0.0001)	—	—
West Africa	0.554 (*p* = 0.093)	0.396 (*p*<0.0001)	0.868 (*p* = 0.663)	0.447 (*p*<0.0001)	0.678 (*p* = 0.014)	0.450 (*p*<0.0001)

The significance was determined by comparison to 10,000 simulated datasets in which the alleles at each locus were reshuffled among all parasites. *p*-Values express the proportion of times the observed genetic distance value was met or exceeded by permutation.

a Populations included in these regions are shown in Figure 5.

## Discussion

We examined the contemporary distribution of *dhps* resistance mutations and found that single, codon 437–mutant (AGK/SGK) alleles were predominant in west and central Africa while the double, codon 437– and 540–mutant (SGE) alleles prevailed throughout east Africa. Flanking sequence analysis showed multiple origins of both single- and double-mutant alleles: three major AGK/SGK lineages and two major SGE lineages. All had been subject to recent selection and each had a highly distinctive regional geographic distribution. In southeast Africa SGE 1 was predominant, while in Northeast Africa SGE 2 prevailed. The three AGK/SGK lineages predominated in different regions: AGK/SGK1 in the southwest, AGK/SGK2 in west Africa, and AGK/SGK3 in central Africa (Cameroon).

### Explaining the Geographical Distributions

The difference between east and west African parasites can most economically be explained as a consequence of the limited number of resistant *dhps* lineages. Each emerged in distinct geographical foci and subsequently became dispersed across a wide region. It has previously been observed with reference to pyrimethamine and chloroquine that the rate of emergence of mutant parasite lineages is far less frequent than might be predicted based on the mutation rate and the number of parasites in each human infection [1], and our findings in relation to the origins of resistant *dhps* are consistent with this observation.

A review of the literature indicates that emergence of all the major resistance lineages took place in the early- to mid-1990s. The A437G substitution was widespread throughout west and central Africa by 1995, reported at prevalences of 37% (27 of 72) in Cameroon in 1995 [42], 28% (10 of 36) in Gabon in 1995 [24], 25% (12 of 48) in Mali 1995 [43], and 25% (8 of 32) in Mali again in 1995 [12]. The distribution of these early observations could imply that all three of the AGK/SGK lineages had emerged and increased in frequency to detectable levels prior to 1995, although it will be necessary for flanking microsatellite analysis to be performed on archived samples to establish this with certainty. The predominance of AGK/SGK lineages hints at their region-specific origins in southwest, west, or central Africa, but the extent of allele sharing between regions demonstrates that parasite migration across these large geographic distances in the intervening years was extensive.

Molecular studies suggest the emergence of SGE 1 and SGE 2 lineages in east Africa also occurred in the mid 1990s. SGE was first reported in samples collected in Kenya during 1993–1995 [36], in Tanzania in 1995 [44], and Malawi in 1995–1996 [12] while in KwaZulu-Natal, South Africa it was absent in 1995–1996 but had appeared by 1999 [13]. We found only the SGE 1 lineage in these countries and infer therefore that these reports describe the emergence of that lineage. The emergence of the SGE 2 lineage occurred in Ethiopia and northeastern Sudan at around the same time. A time series of *dhps* analyses in northeastern Sudan shows the SGE was absent in 1993 but had appeared by 1998 [45]. Although there are no molecular studies of *dhps* in Ethiopia in the same era, three studies in 2004 at widely dispersed geographical sites (this study and [38], [39]) all found that the allele was fixed or almost fixed. This indicates that it had been subject to strong drug selection pressure and perhaps that Ethiopia was the site of its first emergence. Ethiopia officially changed the first-line treatment to SP in 1999 but prior to that SP was widely used for treatment for at least 5 years.

Contrasting with the situation in West and Central Africa we found no mixing of SGE1 and SGE2 between the sites sampled in northeast and southeast Africa. Although the *P. falciparum* populations of Africa are often considered a continuous and largely homogeneous whole, it is likely that there are geospecific factors that promote or restrict the dispersal of mutations through migration of parasites. In this case it seems likely that political instability and civil war placed greater restrictions on travel across conflict zones during this period of *dhps* dispersal. More detailed spatial genetic analysis on the margins of lineage distributions would provide a more precise indication of the forces that govern the dispersal of resistance in these areas.

There was also a marked transition between east and west Africa where the margins of the SGE and AGK/SGK lineage distributions meet. High-resolution mapping of parasite genetics would be valuable for understanding the dynamics underlying these observations. A previous study that compared parasites from multiple sites in DRC during 2003–2004 [46] found the prevalence of K540E was 13.3%–19.3% in eastern DRC but declined to 0.9%–3.9% in western DRC, indicating restricted population mobility between east and west, in this case, undoubtedly exacerbated by a longstanding war during 1998–2003.

This snapshot of the emergence and dispersal of resistant *dhps* within Africa provides an interesting counterpoint to the inferred histories of mutant *pfcrt* and *dhfr* which emerged in Africa some 10–20 years earlier. In those cases a single highly resistant lineage was imported to Africa from Asia and became established in populations throughout the continent. Since the emergence of resistant *dhps* is more recent, it could be argued that the observed distribution of *dhps* resistance alleles is transitional and that given the equivalent amount of time under selection just one resistance lineage would eventually predominate. The SGE 1 lineage is already found in small numbers in Cameroon, Ghana, Guinea, Namibia, Gabon, and Congo, but not in Angola, The Gambia, Mauritania, Mali, Senegal, or Cote d'Ivoire (or Nigeria in this study, although another study has reported the occurrence of 540E in Nigeria) [47]. It is possible that if strong selection through heavy reliance on SP were continued, these foci of SGE would expand, and eventually displace AGK/SGK. The resulting picture would then perhaps be similar to *dhfr*. There are mildly resistant *dhfr* double-mutant lineages believed to be of African origin that were shown to be displaced and outcompeted by the Asian-derived triple-mutant allele [13], [18], with the result that one highly resistant lineage now prevails almost everywhere.

Could migration between east Africa and Asia explain the introgression of SGE alleles in east Africa? Analysis of microsatellites flanking *dhps* in Southeast Asian parasites has not been published but studies in Bangladesh [48] and India [49] show that the SGK/AGK and SGE haplotypes are both common, with SGK/AGK in the majority. This contrasts with the situation in east Africa where SGK/AGK was rare or absent, even before the SGE became so highly prevalent. There is therefore no compelling evidence that the SGE in east Africa is due to extensive parasite exchange between east Africa and Asia. It is noteworthy, however, that we observed an exact match of our SGE 1 flanking haplotype with that of our Southeast Asian control (K1) (details are in Table S2). It is clear that global mapping of *dhps* resistance lineages is needed and the ongoing efforts by local investigators and by the wider research community to assemble a global geography of drug resistance will undoubtedly shed further light on this question [50].

### Parasite Dispersal through Human Migration

The distribution of resistance alleles highlights the importance of human migration in dispersing resistance and parasite infection generally. The regions we defined here on the basis of *dhps* resistance allele-sharing strongly suggest that the economic and transport infrastructures may indirectly govern movement of parasites in Africa through their influence upon volumes of human migration. The regions of allele sharing broadly correspond to African economic communities, which were established to facilitate and promote trade. It would be interesting to explore the contribution of the migrant work force to the dispersal of specific lineages. For example, economic agreements between Gabon and Senegal during the early to mid 1990s were favourable to migrant workers [51], provides a possible explanation for the moderately high frequencies of the AGK/SGK 1 in Senegal, and higher frequencies of AGK/SGK 2 in Gabon compared to its southwest African neighbours.

The first eradication campaigns showed that movement of malaria parasites by human migration can quickly undermine the successful interruption of transmission [52]. Therefore, the impact of control interventions will always be maximised when applied at a geographical scale that encompasses regions of significant volumes of parasite exchange. In the new era of elimination it will be important to understand the forces that govern parasite migration. Dispersal patterns of drug resistance mutations followed in real time can uniquely illustrate the extent and direction of contemporary parasite migration, and further mapping of dispersal of resistance mutations at other loci across Africa, for example the double-mutant *dhfr* lineages which are believed to have emerged de novo in Africa, could confirm the definition of regions of significant parasite exchange. In that context, coordinated campaigns within economic areas such as the Southern African Development Community (SADC) will be more likely to succeed than campaigns within defined national territories that will face an uphill struggle against importation of malaria.

### Implications for Prevention and Treatment

In regions where the *dhps* mutant lineages converged upon the same mutant *dhps* haplotype, we would predict that the SP resistance phenotype of parasites will be equivalent, provided co-adaptive changes at other loci such as *dhfr* or GTP-cyclohydrolase 1 (*gch1*) [53] are also the same. In Sudan and Ethiopia, where we found the SGE 2 lineage, there are also significant differences in the common point mutation haplotypes at *dhfr* [38], [54], [55], and a suggestion that there may be a different SP resistance phenotype [55]. Of note, in-vitro studies [10], [56] indicate that there is higher drug tolerance in double-mutant parasites. To establish whether the different resistance genotypes that predominate in the different geographical regions require different approaches to clinical care, a direct comparison of their drug tolerance levels in vitro as well as in practice is needed. In the absence of such data, evaluation of antifolate-based interventions should be carried out in each of the five lineage-defined geographic regions.

In particular, although growing resistance problems have led to the withdrawal of SP as a first-line treatment for malaria, it is still currently recommended in combination with artesunate in areas where SP resistance is low, and as monotherapy for use as Intermittent Preventive Treatment of Malaria in Pregnancy (IPTp) in all malaria-endemic countries in sub-Saharan Africa [57]. There is strong evidence of a continuing efficacy of SP for IPTp even in areas where SP resistance is well established [58], and SP is also being investigated for use as Intermittent Preventive Treatment of Malaria in Infants (IPTi) [59]. In light of our findings, the continuing assessment of SP efficacy in IPTi, IPTp, and/or clinical treatments that include SP or any other antifolate should be carried out in both east and west African sites.

### Limitations

This study provides a snapshot of the geographical distributions of drug resistance alleles, but these distributions are not static, and it will be important to continue monitoring. By systematic sampling around the boundaries of lineage distributions and in those countries not included so far, a more complete picture of *dhps* allele distribution can be generated. This information will improve our understanding of the true constraints on dispersal of *dhps* mutant alleles at the extremes of the geographical distributions outlined here. It is perhaps surprising that so few studies have attempted to synthesise a geographical analysis of genetic variation in African *P. falciparum*. One difficulty is that research tends to be concentrated into a small number of very well-characterised sites. Another obstacle, when collating data from the published literature, is the absence of a standardised reporting format. In our review of molecular drug resistance data there were many cases in which point mutation data were not presented in a way that allowed the inference of haplotypes—a necessary precondition for inclusion in our analysis. We support the development of a programme to standardise reporting with reference to well-characterised controls, decrease the lag between sampling and publication, and increase the availability of drug resistance data as proposed in the establishment of WARN (World Wide Antimalarial Resistance Network) [50].

### Conclusion

The global movement of resistant malaria has played a decisive role in the establishment of both CQ and pyrimethamine resistance in Africa [1]–[3], but there are significant gaps in our knowledge of when, where, and how resistant genotypes became established on the African continent. Defining the forces that govern the dispersal and successful establishment of resistance genes in Africa is a significant challenge, but will be imperative if emergent resistance to new drug treatments or vaccines is to be managed effectively.

## Supporting Information

Table S1 Frequencies of *dhps* alleles for sites shown in Figure 2.
(0.04 MB PDF)

Table S2 Microsatellite allele data.
(0.08 MB PDF)

Text S1 Details of samples used in this study, including informed consent and ethical approval.
(0.11 MB DOC)

Text S2 Literature search strategy and terms.
(0.03 MB DOC)

## Abbreviations

CQ: chloroquine
CQR: chloroquine resistance
IPTi: Intermittent Preventive Treatment of Malaria in Infants
IPTp: Intermittent Preventive Treatment of Malaria in Pregnancy
SP: sulphadoxine–pyrimethamine
