# Supplementary material for: Multiple Origins and Regional Dispersal of Resistant dhps in African Plasmodium falciparum Malaria
Source: PLoS Med. 2009 Apr 14;6(4):e1000055. doi: 10.1371/journal.pmed.1000055 (PMC2661256; doi:10.1371/journal.pmed.1000055)
Supplement: Table S1 — Frequencies of dhps alleles for sites shown in Figure 2. (0.04 MB PDF) [file pmed.1000055.s001.pdf]

Country	Study Site	Year	Region	Longitude	Latitude	n	SAK	AAK	SGK	SGE	AGK	Rare Alleles	Mixed	TOTAL
Cameroon	Garoua	2004	Central	13.40	9.30	71	10	42	9	0	7	3	26	97
	Mutengene	2004	Central	9.31	4.10	202	6	22	148	1	21	4	25	227
	Yaounde	2004	Central	11.51	3.86	143	10	41	64	0	25	3	36	179
Central African Republic	Bangui	2004	Central	21.00	7.00	74	7	55	6	1	5	0	n/a	
Congo	Pointe Noire & Brazzaville	2002	Central	11.83	-4.79	135	32	12	75	0	16	0	n/a	
	Kindamba	2004	Central	15.05	-4.30	236	44	20	164	2	5	1	20	256
Equatorial Guinea			Central	10.00	2.00	12	2	1	7	0	1	1	0	12
Gabon	Haut-Ogooue	2000	Central	13.58	-1.63	82	8	22	46	0	6	0	n/a	
	Lambarene	2007	Central	10.21	-0.71	64	1	15	38	4	6	0	10	74
DRC	Shabunda	2004	East	27.33	-2.70	177	53	43	1	80	0	0	29	206
Kenya	Bondo	2006	East	34.27	0.24	133	1	0	4	127	0	1	13	146
Malawi	Salima	2000	East	34.50	-13.75	159	7	0	0	141	0	11	8	167
Tanzania	Hai	2001	East	37.17	-3.17	81	23	6	0	52	0	0	5	86
	N.Pare	2001	East	37.67	-3.75	30	16	1	0	13	0	0	5	35
	S.Pare	2001	East	37.87	-4.25	33	13	2	0	18	0	0	9	42
	Kilombero & Ulanga	2004	East	36.68	-8.14	561	202	30	5	317	0	7	152	713
Uganda	Kabale & Rukungiri	2005	East	30.00	-1.26	127	1	0	0	126	0	0	5	132
Ethiopia	Dilla	2004	Northeast	38.32	6.42	69	2	0	0	67	0	0	0	69
	Humera	2004	Northeast	36.66	14.32	87	1	0	1	85	0	0	2	89
	Jimma	2004	Northeast	36.83	7.67	124	0	0	0	122	0	2	0	124
Sudan	Lankien	2001	Northeast	32.03	8.48	44	44	0	0	0	0	0	0	44
	Yargot Payam Bahr el Gazal	2002	Northeast	27.50	8.98	75	35	33	0	7	0	0	0	75
	Gedaref	2003	Northeast	35.38	14.04	69	0	0	1	68	0	0	2	71
Mozambique	East Rural	2001	Southeast	32.73	-26.57	110	86	2	0	21	1	0	8	118
	Peri-Urban	2001	Southeast	32.48	-25.96	134	92	7	1	34	0	0	0	134
	West Rural	2001	Southeast	32.28	-26.42	96	75	4	2	15	0	0	11	107
South Africa	Ingwavuma	1999	Southeast	31.98	-27.13	198	140	3	2	53	0	0	56	254
	Komatipoort	2000	Southeast	31.93	-25.43	306	258	18	0	30	0	0	1	307
Zambia	Chibombo	2004	Southeast	28.07	-14.65	15	4	0	0	10	0	1	1	17
	Chipata	2004	Southeast	31.93	-11.50	12	3	0	0	9	0	0	0	12
	Chongwe	2004	Southeast	32.65	-13.80	58	26	1	0	29	0	2	5	63
	Isoka	2004	Southeast	32.63	-10.13	54	13	1	1	37	0	2	13	54
	Mansa	2004	Southeast	28.88	-11.20	22	9	0	1	12	0	0	1	23
	Mpongwe	2004	Southeast	28.15	-13.53	24	12	0	1	11	0	0	2	27
Angola	Uige Province	2004	Southwest	15.07	-7.62	40	2	0	15	0	22	1	9	49
Namibia	Kavango	2005	Southwest	17.08	-22.57	76	10	0	50	7	9	0	7	83
Burkina Faso	Bousse	2002	West	-1.60	12.60	365	21	99	56	0	153	36	69	434
	Nanoro	2003	West	-2.20	12.68	60	1	0	25	0	34	0	n/a	
Cote d'Ivoire	Yopougon, Abidjan	2001	West	-4.07	5.36	118	7	27	32	0	14	38	0	118
Gambia	Farafenni	2004	West	-15.60	13.57	127	47	18	57	0	2	3	29	156
Ghana	Navarongo	2003	West	-1.09	10.90	101	0	12	29	1	57	2	52	153
	Hoehoe	2005	West	0.47	6.60	126	1	2	49	1	70	3	45	171
Guinea	Laine	2004	West	-8.48	8.08	114	8	31	38	8	29	0	21	135
Guinea Bissau	Bandim		West	-15.38	11.34	91	48	19	21	0	3	0	6	97
Mali		1997	West	-7.93	12.65	13	0	0	0	0	0	13	5	18
Mauritania	Aioun & Kobeni	1998	West	-10.00	16.50	160	58	66	19	0	10	7	0	160
Nigeria	Abuja	2005	West	7.53	9.08	17	3	5	6	0	2	1	0	17
Senegal	Pikine	2003	West	-17.40	14.75	15	7	2	5	0	1	0	0	15
	Niakar	2004	West	-16.40	14.48	234	75	39	116	0	2	2	151	385
