# Supplementary material for: Multiple Origins and Regional Dispersal of Resistant dhps in African Plasmodium falciparum Malaria
Source: PLoS Med. 2009 Apr 14;6(4):e1000055. doi: 10.1371/journal.pmed.1000055 (PMC2661256; doi:10.1371/journal.pmed.1000055)
Supplement: Table S2 — Microsatellite allele data. (0.08 MB PDF) [file pmed.1000055.s002.pdf]

Population	N (samples preselected for AAK, SAK, SGE, SGK, AGK alleles)	Samples lost to analysis	N
Angola	39	0	39
Burkina Faso	116	16	100
Cameroon Mutengene	198	15	183
Cameroon Yaounde	105	7	98
Congo	158	4	154
DRC	73	6	67
Ethiopia	38	0	38
Gabon	64	2	62
Ghana	106	11	95
Guinea	59	3	56
Kenya	112	1	111
Mozambique	136	26	110
Namibia	76	1	75
Nigeria	16	1	15
Senegal	45	1	44
South Africa	27	0	27
Sudan	69	1	68
Tanzania	105	16	89
Uganda	133	4	129
Zambia	119	5	114
	1794	120	1674

Population ID	DHPS	0.8kb	4.3kb	7.7kb	2 locus haplotype code	Lineage code
Angola	AGK	117	104	119	H21	AGK/SGK1
Angola	AGK	117	106	103	H22	AGK/SGK1
Angola	AGK	117	106	111	H22	AGK/SGK1
Angola	AGK	117	106	117	H22	AGK/SGK1
Angola	AGK	117	106	125	H22	AGK/SGK1
Angola	AGK	117	106	141	H22	AGK/SGK1
Angola	AGK	117	106	141	H22	AGK/SGK1
Angola	AGK	117	106	141	H22	AGK/SGK1
Angola	AGK	117	106	141	H22	AGK/SGK1
Angola	AGK	117	106	141	H22	AGK/SGK1
Angola	AGK	117	106	141	H22	AGK/SGK1
Angola	AGK	117	106	141	H22	AGK/SGK1
Angola	AGK	117	106	141	H22	AGK/SGK1
Angola	AGK	117	106	141	H22	AGK/SGK1
Angola	AGK	117	106	-	H22	AGK/SGK1
Angola	AGK	117	106	-	H22	AGK/SGK1
Angola	AGK	117	106	-	H22	AGK/SGK1
Angola	AGK	117	106	-	H22	AGK/SGK1
Angola	AGK	117	106	-	H22	AGK/SGK1
Angola	AGK	117	106	-	H22	AGK/SGK1
Angola	AGK	117	106	-	H22	AGK/SGK1
Angola	AGK	117	106	-	H22	AGK/SGK1
Angola	SAK	117	104	111	H21	
Angola	SAK	-	106	113		
Angola	SGK	117	106	111	H22	AGK/SGK1
Angola	SGK	117	106	113	H22	AGK/SGK1
Angola	SGK	117	106	119	H22	AGK/SGK1
Angola	SGK	117	106	121	H22	AGK/SGK1
Angola	SGK	117	106	127	H22	AGK/SGK1
Angola	SGK	117	106	141	H22	AGK/SGK1
Angola	SGK	117	106	141	H22	AGK/SGK1
Angola	SGK	117	106	141	H22	AGK/SGK1
Angola	SGK	117	106	143	H22	AGK/SGK1
Angola	SGK	117	106	-	H22	AGK/SGK1
Angola	SGK	117	106	-	H22	AGK/SGK1
Angola	SGK	117	106	-	H22	AGK/SGK1
Angola	SGK	117	106	-	H22	AGK/SGK1
Angola	SGK	-	106	143		
Angola	SGK	-	106	-		
BurkinaFaso	AAK	105	-	119		
BurkinaFaso	AAK	105	-	-		
BurkinaFaso	AAK	105	-	-		
BurkinaFaso	AAK	107	112	117	H06	

Population ID	DHPS	0.8kb	4.3kb	7.7kb	2 locus haplotype code	Lineage code
BurkinaFaso	AAK	115	106	-	H15	
BurkinaFaso	AAK	115	116	117	H19	
BurkinaFaso	AAK	115	-	113		
BurkinaFaso	AAK	115	-	-		
BurkinaFaso	AAK	115	-	-		
BurkinaFaso	AAK	115	-	-		
BurkinaFaso	AAK	115	-	-		
BurkinaFaso	AAK	119	104	117	H27	
BurkinaFaso	AAK	119	-	-		
BurkinaFaso	AAK	121	108	113	H34	
BurkinaFaso	AAK	121	112	119	H36	
BurkinaFaso	AAK	121	-	-		
BurkinaFaso	AAK	121	-	-		
BurkinaFaso	AAK	123	106	113	H43	
BurkinaFaso	AAK	123	106	113	H43	
BurkinaFaso	AAK	125	-	-		
BurkinaFaso	AAK	127	106	123	H55	
BurkinaFaso	AAK	129	108	119	H61	
BurkinaFaso	AAK	-	106	113		
BurkinaFaso	AAK	-	106	117		
BurkinaFaso	AAK	-	106	123		
BurkinaFaso	AAK	-	108	113		
BurkinaFaso	AAK	-	108	-		
BurkinaFaso	AAK	-	-	113		
BurkinaFaso	AAK	-	-	113		
BurkinaFaso	AAK	-	-	113		
BurkinaFaso	AAK	-	-	113		
BurkinaFaso	AAK	-	-	113		
BurkinaFaso	AAK	-	-	113		
BurkinaFaso	AAK	-	-	117		
BurkinaFaso	AAK	-	-	119		
BurkinaFaso	AGK	107	106	113	H05	
BurkinaFaso	AGK	115	108	115	H16	
BurkinaFaso	AGK	115	116	117	H19	
BurkinaFaso	AGK	115	-	-		
BurkinaFaso	AGK	121	108	113	H34	AGK/SGK2
BurkinaFaso	AGK	121	108	113	H34	AGK/SGK2
BurkinaFaso	AGK	121	108	113	H34	AGK/SGK2
BurkinaFaso	AGK	121	108	113	H34	AGK/SGK2
BurkinaFaso	AGK	121	108	113	H34	AGK/SGK2
BurkinaFaso	AGK	121	108	113	H34	AGK/SGK2
BurkinaFaso	AGK	121	108	113	H34	AGK/SGK2
BurkinaFaso	AGK	121	108	113	H34	AGK/SGK2

Population ID	DHPS	0.8kb	4.3kb	7.7kb	2 locus haplotype code	Lineage code
BurkinaFaso	AGK	121	108	115	H34	AGK/SGK2
BurkinaFaso	AGK	121	112	119	H36	AGK/SGK2
BurkinaFaso	AGK	121	112	-	H36	AGK/SGK2
BurkinaFaso	AGK	121	-	113		
BurkinaFaso	AGK	121	-	113		
BurkinaFaso	AGK	121	-	113		
BurkinaFaso	AGK	121	-	113		
BurkinaFaso	AGK	121	-	113		
BurkinaFaso	AGK	121	-	-		
BurkinaFaso	AGK	121	-	-		
BurkinaFaso	AGK	121	-	-		
BurkinaFaso	AGK	127	106	113	H55	
BurkinaFaso	AGK	127	106	113	H55	
BurkinaFaso	AGK	127	106	113	H55	
BurkinaFaso	AGK	127	108	113	H56	
BurkinaFaso	AGK	-	106	113		
BurkinaFaso	AGK	-	106	113		
BurkinaFaso	AGK	-	106	113		
BurkinaFaso	AGK	-	106	-		
BurkinaFaso	AGK	-	108	113		
BurkinaFaso	AGK	-	108	113		
BurkinaFaso	AGK	-	-	113		
BurkinaFaso	AGK	-	-	113		
BurkinaFaso	AGK	-	-	113		
BurkinaFaso	AGK	-	-	113		
BurkinaFaso	AGK	-	-	117		
BurkinaFaso	SAK	103	-	117		
BurkinaFaso	SAK	119	104	117	H27	
BurkinaFaso	SAK	119	-	-		
BurkinaFaso	SAK	119	-	-		
BurkinaFaso	SAK	123	-	-		
BurkinaFaso	SAK	-	104	113		
BurkinaFaso	SAK	-	108	113		
BurkinaFaso	SGK	105	116	-	H04	
BurkinaFaso	SGK	115	116	113	H19	
BurkinaFaso	SGK	115	-	117		
BurkinaFaso	SGK	121	108	113	H34	AGK/SGK2
BurkinaFaso	SGK	121	108	113	H34	AGK/SGK2
BurkinaFaso	SGK	121	108	113	H34	AGK/SGK2
BurkinaFaso	SGK	121	108	117	H34	AGK/SGK2
BurkinaFaso	SGK	121	116	113	H38	
BurkinaFaso	SGK	121	-	113		
BurkinaFaso	SGK	121	-	-		

Population ID	DHPS	0.8kb	4.3kb	7.7kb	2 locus haplotype code	Lineage code
BurkinaFaso	SGK	123	-	113		
BurkinaFaso	SGK	123	-	-		
BurkinaFaso	SGK	129	104	113	H59	
BurkinaFaso	SGK	129	-	125		
BurkinaFaso	SGK	-	100	-		
BurkinaFaso	SGK	-	108	113		
BurkinaFaso	SGK	-	108	113		
BurkinaFaso	SGK	-	108	-		
BurkinaFaso	SGK	-	110	129		
BurkinaFaso	SGK	-	-	127		
Cameroon Mutengene	AAK	109	108	109	H07	
Cameroon Mutengene	AAK	115	106	117	H15	
Cameroon Mutengene	AAK	123	108	109	H44	
Cameroon Mutengene	AAK	125	104	123	H50	
Cameroon Mutengene	AAK	125	110	119	H53	
Cameroon Mutengene	AAK	129	118	125	H63	
Cameroon Mutengene	AAK	131	118	125	H73	
Cameroon Mutengene	AAK	131	118	125	H73	
Cameroon Mutengene	AAK	131	118	125	H73	
Cameroon Mutengene	AAK	-	104	115		
Cameroon Mutengene	AAK	-	106	125		
Cameroon Mutengene	AAK	-	108	-		
Cameroon Mutengene	AAK	-	110	113		
Cameroon Mutengene	AAK	-	118	125		
Cameroon Mutengene	AAK	-	118	125		
Cameroon Mutengene	AAK	-	-	113		
Cameroon Mutengene	AAK	-	-	125		
Cameroon Mutengene	AAK	-	-	125		
Cameroon Mutengene	AGK	109	108	109	H07	
Cameroon Mutengene	AGK	113	106	113	H12	
Cameroon Mutengene	AGK	115	104	-	H15	
Cameroon Mutengene	AGK	117	106	-	H22	AGK/SGK1
Cameroon Mutengene	AGK	121	104	111	H32	AGK/SGK2
Cameroon Mutengene	AGK	121	108	113	H34	AGK/SGK2
Cameroon Mutengene	AGK	121	110	111	H35	AGK/SGK2
Cameroon Mutengene	AGK	121	110	111	H35	AGK/SGK2
Cameroon Mutengene	AGK	121	110	111	H35	AGK/SGK2
Cameroon Mutengene	AGK	121	110	127	H35	AGK/SGK2
Cameroon Mutengene	AGK	121	110	-	H35	AGK/SGK2
Cameroon Mutengene	AGK	123	108	123	H44	AGK/SGK3
Cameroon Mutengene	AGK	125	104	119	H50	
Cameroon Mutengene	AGK	125	104	119	H50	
Cameroon Mutengene	AGK	125	110	125	H53	

Population ID	DHPS	0.8kb	4.3kb	7.7kb	2 locus haplotype code	Lineage code
Cameroon Mutengene	AGK	131	-	115		
Cameroon Mutengene	AGK	-	106	-		
Cameroon Mutengene	AGK	-	110	117		
Cameroon Mutengene	AGK	-	-	117		
Cameroon Mutengene	SAK	117	104	-	H21	
Cameroon Mutengene	SAK	119	104	105	H27	
Cameroon Mutengene	SAK	-	108	109		
Cameroon Mutengene	SAK	-	108	115		
Cameroon Mutengene	SAK	-	-	107		
Cameroon Mutengene	SGE	-	104	107		
Cameroon Mutengene	SGK	111	104	111	H08	
Cameroon Mutengene	SGK	111	112	123	H10	
Cameroon Mutengene	SGK	115	106	-	H15	
Cameroon Mutengene	SGK	117	106	-	H22	AGK/SGK1
Cameroon Mutengene	SGK	117	108	109	H23	AGK/SGK1
Cameroon Mutengene	SGK	117	110	109	H24	AGK/SGK1
Cameroon Mutengene	SGK	117	110	123	H24	AGK/SGK1
Cameroon Mutengene	SGK	119	106	109	H28	
Cameroon Mutengene	SGK	119	108	117	H29	
Cameroon Mutengene	SGK	121	104	111	H32	AGK/SGK2
Cameroon Mutengene	SGK	121	106	-	H33	AGK/SGK2
Cameroon Mutengene	SGK	121	108	109	H34	AGK/SGK2
Cameroon Mutengene	SGK	121	108	109	H34	AGK/SGK2
Cameroon Mutengene	SGK	121	108	113	H34	AGK/SGK2
Cameroon Mutengene	SGK	121	110	111	H35	AGK/SGK2
Cameroon Mutengene	SGK	121	110	111	H35	AGK/SGK2
Cameroon Mutengene	SGK	121	110	111	H35	AGK/SGK2
Cameroon Mutengene	SGK	121	116	113	H38	
Cameroon Mutengene	SGK	121	-	111		
Cameroon Mutengene	SGK	121	-	113		
Cameroon Mutengene	SGK	121	-	113		
Cameroon Mutengene	SGK	123	106	109	H43	AGK/SGK3
Cameroon Mutengene	SGK	123	106	119	H43	AGK/SGK3
Cameroon Mutengene	SGK	123	106	-	H43	AGK/SGK3
Cameroon Mutengene	SGK	123	108	109	H44	AGK/SGK3
Cameroon Mutengene	SGK	123	108	109	H44	AGK/SGK3
Cameroon Mutengene	SGK	123	108	109	H44	AGK/SGK3
Cameroon Mutengene	SGK	123	108	109	H44	AGK/SGK3
Cameroon Mutengene	SGK	123	108	109	H44	AGK/SGK3
Cameroon Mutengene	SGK	123	108	109	H44	AGK/SGK3
Cameroon Mutengene	SGK	123	108	109	H44	AGK/SGK3
Cameroon Mutengene	SGK	123	108	109	H44	AGK/SGK3
Cameroon Mutengene	SGK	123	108	109	H44	AGK/SGK3

Population ID	DHPS	0.8kb	4.3kb	7.7kb	2 locus haplotype code	Lineage code
Cameroon Mutengene	SGK	123	108	109	H44	AGK/SGK3
Cameroon Mutengene	SGK	123	108	109	H44	AGK/SGK3
Cameroon Mutengene	SGK	123	108	109	H44	AGK/SGK3
Cameroon Mutengene	SGK	123	108	109	H44	AGK/SGK3
Cameroon Mutengene	SGK	123	108	109	H44	AGK/SGK3
Cameroon Mutengene	SGK	123	108	109	H44	AGK/SGK3
Cameroon Mutengene	SGK	123	108	109	H44	AGK/SGK3
Cameroon Mutengene	SGK	123	108	109	H44	AGK/SGK3
Cameroon Mutengene	SGK	123	108	109	H44	AGK/SGK3
Cameroon Mutengene	SGK	123	108	109	H44	AGK/SGK3
Cameroon Mutengene	SGK	123	108	109	H44	AGK/SGK3
Cameroon Mutengene	SGK	123	108	109	H44	AGK/SGK3
Cameroon Mutengene	SGK	123	108	109	H44	AGK/SGK3
Cameroon Mutengene	SGK	123	108	109	H44	AGK/SGK3
Cameroon Mutengene	SGK	123	108	109	H44	AGK/SGK3
Cameroon Mutengene	SGK	123	108	109	H44	AGK/SGK3
Cameroon Mutengene	SGK	123	108	109	H44	AGK/SGK3
Cameroon Mutengene	SGK	123	108	109	H44	AGK/SGK3
Cameroon Mutengene	SGK	123	108	109	H44	AGK/SGK3
Cameroon Mutengene	SGK	123	108	109	H44	AGK/SGK3
Cameroon Mutengene	SGK	123	108	109	H44	AGK/SGK3
Cameroon Mutengene	SGK	123	108	109	H44	AGK/SGK3
Cameroon Mutengene	SGK	123	108	109	H44	AGK/SGK3
Cameroon Mutengene	SGK	123	108	109	H44	AGK/SGK3
Cameroon Mutengene	SGK	123	108	109	H44	AGK/SGK3
Cameroon Mutengene	SGK	123	108	109	H44	AGK/SGK3
Cameroon Mutengene	SGK	123	108	111	H44	AGK/SGK3
Cameroon Mutengene	SGK	123	108	119	H44	AGK/SGK3
Cameroon Mutengene	SGK	123	108	123	H44	AGK/SGK3
Cameroon Mutengene	SGK	123	108	129	H44	AGK/SGK3
Cameroon Mutengene	SGK	123	108	-	H44	AGK/SGK3
Cameroon Mutengene	SGK	123	108	-	H44	AGK/SGK3
Cameroon Mutengene	SGK	123	108	-	H44	AGK/SGK3
Cameroon Mutengene	SGK	123	108	-	H44	AGK/SGK3
Cameroon Mutengene	SGK	123	118	121	H48	
Cameroon Mutengene	SGK	123	-	109		
Cameroon Mutengene	SGK	123	-	109		
Cameroon Mutengene	SGK	123	-	109		
Cameroon Mutengene	SGK	123	-	-		
Cameroon Mutengene	SGK	123	-	-		
Cameroon Mutengene	SGK	123	-	-		
Cameroon Mutengene	SGK	125	104	-	H50	
Cameroon Mutengene	SGK	125	108	109	H52	

Population ID	DHPS	0.8kb	4.3kb	7.7kb	2 locus haplotype code	Lineage code
Cameroon Mutengene	SGK	125	108	109	H52	
Cameroon Mutengene	SGK	125	108	125	H52	
Cameroon Mutengene	SGK	125	-	-		
Cameroon Mutengene	SGK	129	106	113	H60	
Cameroon Mutengene	SGK	131	106	113	H67	
Cameroon Mutengene	SGK	133	104	127	H76	
Cameroon Mutengene	SGK	136	104	119	H81	
Cameroon Mutengene	SGK	141	106	119	H88	
Cameroon Mutengene	SGK	-	104	111		
Cameroon Mutengene	SGK	-	104	111		
Cameroon Mutengene	SGK	-	104	111		
Cameroon Mutengene	SGK	-	104	115		
Cameroon Mutengene	SGK	-	104	117		
Cameroon Mutengene	SGK	-	104	119		
Cameroon Mutengene	SGK	-	104	-		
Cameroon Mutengene	SGK	-	106	113		
Cameroon Mutengene	SGK	-	106	113		
Cameroon Mutengene	SGK	-	106	113		
Cameroon Mutengene	SGK	-	106	119		
Cameroon Mutengene	SGK	-	106	119		
Cameroon Mutengene	SGK	-	106	119		
Cameroon Mutengene	SGK	-	106	119		
Cameroon Mutengene	SGK	-	106	119		
Cameroon Mutengene	SGK	-	106	-		
Cameroon Mutengene	SGK	-	106	-		
Cameroon Mutengene	SGK	-	106	-		
Cameroon Mutengene	SGK	-	108	109		
Cameroon Mutengene	SGK	-	108	109		
Cameroon Mutengene	SGK	-	108	109		
Cameroon Mutengene	SGK	-	108	109		
Cameroon Mutengene	SGK	-	108	109		
Cameroon Mutengene	SGK	-	108	109		
Cameroon Mutengene	SGK	-	108	109		
Cameroon Mutengene	SGK	-	108	109		
Cameroon Mutengene	SGK	-	108	109		
Cameroon Mutengene	SGK	-	108	109		
Cameroon Mutengene	SGK	-	108	109		
Cameroon Mutengene	SGK	-	108	109		
Cameroon Mutengene	SGK	-	108	109		
Cameroon Mutengene	SGK	-	108	109		
Cameroon Mutengene	SGK	-	108	109		
Cameroon Mutengene	SGK	-	108	117		
Cameroon Mutengene	SGK	-	108	117		

Population ID	DHPS	0.8kb	4.3kb	7.7kb	2 locus haplotype code	Lineage code
Cameroon Mutengene	SGK	-	108	119		
Cameroon Mutengene	SGK	-	108	-		
Cameroon Mutengene	SGK	-	108	-		
Cameroon Mutengene	SGK	-	108	-		
Cameroon Mutengene	SGK	-	108	-		
Cameroon Mutengene	SGK	-	116	-		
Cameroon Mutengene	SGK	-	118	127		
Cameroon Mutengene	SGK	-	-	109		
Cameroon Mutengene	SGK	-	-	109		
Cameroon Mutengene	SGK	-	-	109		
Cameroon Mutengene	SGK	-	-	109		
Cameroon Mutengene	SGK	-	-	111		
Cameroon Mutengene	SGK	-	-	111		
Cameroon Mutengene	SGK	-	-	111		
Cameroon Mutengene	SGK	-	-	115		
Cameroon Mutengene	SGK	-	-	115		
Cameroon Mutengene	SGK	-	-	115		
Cameroon Mutengene	SGK	-	-	117		
Cameroon Mutengene	SGK	-	-	119		
Cameroon Mutengene	SGK	-	-	119		
Cameroon Mutengene	SGK	-	-	123		
Cameroon Yaounde	AAK	119	104	105	H27	
Cameroon Yaounde	AAK	123	108	109	H44	
Cameroon Yaounde	AAK	123	-	-		
Cameroon Yaounde	AAK	125	108	109	H52	
Cameroon Yaounde	AAK	127	104	111	H54	
Cameroon Yaounde	AAK	127	106	113	H55	
Cameroon Yaounde	AAK	129	104	123	H59	
Cameroon Yaounde	AAK	131	106	125	H67	
Cameroon Yaounde	AAK	131	118	107	H73	
Cameroon Yaounde	AAK	131	118	125	H73	
Cameroon Yaounde	AAK	131	118	125	H73	
Cameroon Yaounde	AAK	131	118	125	H73	
Cameroon Yaounde	AAK	131	118	125	H73	
Cameroon Yaounde	AAK	131	118	125	H73	
Cameroon Yaounde	AAK	131	118	127	H73	
Cameroon Yaounde	AAK	131	118	-	H73	
Cameroon Yaounde	AAK	131	118	-	H73	
Cameroon Yaounde	AAK	131	-	-		
Cameroon Yaounde	AAK	133	118	125	H80	
Cameroon Yaounde	AAK	147	104	115	H91	
Cameroon Yaounde	AAK	-	108	109		
Cameroon Yaounde	AAK	-	108	-		

Population ID	DHPS	0.8kb	4.3kb	7.7kb	2 locus haplotype code	Lineage code
Cameroon Yaounde	AAK	-	118	125		
Cameroon Yaounde	AGK	117	106	141	H22	AGK/SGK1
Cameroon Yaounde	AGK	117	106	141	H22	AGK/SGK1
Cameroon Yaounde	AGK	117	106	141	H22	AGK/SGK1
Cameroon Yaounde	AGK	117	106	141	H22	AGK/SGK1
Cameroon Yaounde	AGK	117	106	141	H22	AGK/SGK1
Cameroon Yaounde	AGK	117	106	-	H22	AGK/SGK1
Cameroon Yaounde	AGK	117	106	-	H22	AGK/SGK1
Cameroon Yaounde	AGK	117	106	-	H22	AGK/SGK1
Cameroon Yaounde	AGK	117	106	-	H22	AGK/SGK1
Cameroon Yaounde	AGK	117	-	141		
Cameroon Yaounde	AGK	117	-	-		
Cameroon Yaounde	AGK	121	108	113	H34	AGK/SGK2
Cameroon Yaounde	AGK	121	110	111	H35	AGK/SGK2
Cameroon Yaounde	AGK	121	110	-	H35	AGK/SGK2
Cameroon Yaounde	AGK	121	110	-	H35	AGK/SGK2
Cameroon Yaounde	AGK	121	-	111		
Cameroon Yaounde	AGK	121	-	127		
Cameroon Yaounde	AGK	125	104	119	H50	
Cameroon Yaounde	SAK	117	104	129	H21	
Cameroon Yaounde	SAK	117	-	127		
Cameroon Yaounde	SAK	117	-	-		
Cameroon Yaounde	SAK	125	106	127	H51	
Cameroon Yaounde	SAK	125	110	125	H53	
Cameroon Yaounde	SAK	131	110	125	H69	
Cameroon Yaounde	SAK	136	106	125	H82	
Cameroon Yaounde	SAK	145	106	125	H90	
Cameroon Yaounde	SGK	117	106	121	H22	AGK/SGK1
Cameroon Yaounde	SGK	117	110	123	H24	AGK/SGK1
Cameroon Yaounde	SGK	121	108	113	H34	AGK/SGK2
Cameroon Yaounde	SGK	121	108	-	H34	AGK/SGK2
Cameroon Yaounde	SGK	123	104	123	H42	AGK/SGK3
Cameroon Yaounde	SGK	123	106	125	H43	AGK/SGK3
Cameroon Yaounde	SGK	123	108	109	H44	AGK/SGK3
Cameroon Yaounde	SGK	123	108	109	H44	AGK/SGK3
Cameroon Yaounde	SGK	123	108	109	H44	AGK/SGK3
Cameroon Yaounde	SGK	123	108	109	H44	AGK/SGK3
Cameroon Yaounde	SGK	123	108	109	H44	AGK/SGK3
Cameroon Yaounde	SGK	123	108	109	H44	AGK/SGK3
Cameroon Yaounde	SGK	123	108	109	H44	AGK/SGK3
Cameroon Yaounde	SGK	123	108	109	H44	AGK/SGK3
Cameroon Yaounde	SGK	123	108	109	H44	AGK/SGK3
Cameroon Yaounde	SGK	123	108	109	H44	AGK/SGK3

Population ID	DHPS	0.8kb	4.3kb	7.7kb	2 locus haplotype code	Lineage code
Cameroon Yaounde	SGK	123	108	109	H44	AGK/SGK3
Cameroon Yaounde	SGK	123	108	109	H44	AGK/SGK3
Cameroon Yaounde	SGK	123	108	109	H44	AGK/SGK3
Cameroon Yaounde	SGK	123	108	109	H44	AGK/SGK3
Cameroon Yaounde	SGK	123	108	109	H44	AGK/SGK3
Cameroon Yaounde	SGK	123	108	109	H44	AGK/SGK3
Cameroon Yaounde	SGK	123	108	109	H44	AGK/SGK3
Cameroon Yaounde	SGK	123	108	109	H44	AGK/SGK3
Cameroon Yaounde	SGK	123	108	109	H44	AGK/SGK3
Cameroon Yaounde	SGK	123	108	109	H44	AGK/SGK3
Cameroon Yaounde	SGK	123	108	109	H44	AGK/SGK3
Cameroon Yaounde	SGK	123	108	109	H44	AGK/SGK3
Cameroon Yaounde	SGK	123	108	109	H44	AGK/SGK3
Cameroon Yaounde	SGK	123	108	109	H44	AGK/SGK3
Cameroon Yaounde	SGK	123	108	109	H44	AGK/SGK3
Cameroon Yaounde	SGK	123	108	115	H44	AGK/SGK3
Cameroon Yaounde	SGK	123	108	-	H44	AGK/SGK3
Cameroon Yaounde	SGK	123	108	-	H44	AGK/SGK3
Cameroon Yaounde	SGK	123	-	109		
Cameroon Yaounde	SGK	123	-	-		
Cameroon Yaounde	SGK	123	-	-		
Cameroon Yaounde	SGK	125	108	109	H52	
Cameroon Yaounde	SGK	125	108	109	H52	
Cameroon Yaounde	SGK	125	108	125	H52	
Cameroon Yaounde	SGK	125	108	-	H52	
Cameroon Yaounde	SGK	125	110	117	H53	
Cameroon Yaounde	SGK	127	112	113	H57	
Cameroon Yaounde	SGK	131	118	125	H73	
Cameroon Yaounde	SGK	133	104	111	H76	
Cameroon Yaounde	SGK	133	104	111	H76	
Cameroon Yaounde	SGK	133	118	129	H80	
Cameroon Yaounde	SGK	-	106	119		
Cameroon Yaounde	SGK	-	106	-		
Congo	AAK	117	106	109	H22	
Congo	AAK	117	106	115	H22	
Congo	AAK	117	106	123	H22	
Congo	AAK	117	106	141	H22	
Congo	AAK	117	106	141	H22	
Congo	AAK	117	106	-	H22	
Congo	AAK	117	106	-	H22	
Congo	AAK	117	106	-	H22	
Congo	AAK	117	108	-	H23	
Congo	AAK	117	-	111		

Population ID	DHPS	0.8kb	4.3kb	7.7kb	2 locus haplotype code	Lineage code
Congo	AAK	121	108	123	H34	
Congo	AAK	131	102	141	H65	
Congo	AAK	-	106	-		
Congo	AGK	-	106	-		
Congo	SAK	117	106	105	H22	
Congo	SAK	117	106	123	H22	
Congo	SAK	117	106	123	H22	
Congo	SAK	117	106	141	H22	
Congo	SAK	117	106	141	H22	
Congo	SAK	117	106	141	H22	
Congo	SAK	117	106	141	H22	
Congo	SAK	117	106	-	H22	
Congo	SAK	117	106	-	H22	
Congo	SAK	117	108	-	H23	
Congo	SAK	117	-	113		
Congo	SAK	119	116	129	H31	
Congo	SAK	121	104	109	H32	
Congo	SAK	121	108	109	H34	
Congo	SAK	121	108	123	H34	
Congo	SAK	121	110	141	H35	
Congo	SAK	121	-	109		
Congo	SAK	123	108	125	H44	
Congo	SAK	125	104	123	H50	
Congo	SAK	131	108	132	H68	
Congo	SAK	149	104	121	H92	
Congo	SAK	-	104	105		
Congo	SAK	-	106	119		
Congo	SAK	-	110	121		
Congo	SGE	117	106	117	H22	
Congo	SGE	117	106	132	H22	
Congo	SGK	113	106	136	H12	
Congo	SGK	115	106	-	H15	
Congo	SGK	117	104	107	H21	AGK/SGK1
Congo	SGK	117	104	107	H21	AGK/SGK1
Congo	SGK	117	106	103	H22	AGK/SGK1
Congo	SGK	117	106	105	H22	AGK/SGK1
Congo	SGK	117	106	107	H22	AGK/SGK1
Congo	SGK	117	106	109	H22	AGK/SGK1
Congo	SGK	117	106	109	H22	AGK/SGK1
Congo	SGK	117	106	111	H22	AGK/SGK1
Congo	SGK	117	106	111	H22	AGK/SGK1
Congo	SGK	117	106	111	H22	AGK/SGK1
Congo	SGK	117	106	111	H22	AGK/SGK1

Population ID	DHPS	0.8kb	4.3kb	7.7kb	2 locus haplotype code	Lineage code
Congo	SGK	117	106	113	H22	AGK/SGK1
Congo	SGK	117	106	115	H22	AGK/SGK1
Congo	SGK	117	106	115	H22	AGK/SGK1
Congo	SGK	117	106	117	H22	AGK/SGK1
Congo	SGK	117	106	117	H22	AGK/SGK1
Congo	SGK	117	106	117	H22	AGK/SGK1
Congo	SGK	117	106	119	H22	AGK/SGK1
Congo	SGK	117	106	119	H22	AGK/SGK1
Congo	SGK	117	106	119	H22	AGK/SGK1
Congo	SGK	117	106	121	H22	AGK/SGK1
Congo	SGK	117	106	123	H22	AGK/SGK1
Congo	SGK	117	106	123	H22	AGK/SGK1
Congo	SGK	117	106	123	H22	AGK/SGK1
Congo	SGK	117	106	123	H22	AGK/SGK1
Congo	SGK	117	106	123	H22	AGK/SGK1
Congo	SGK	117	106	123	H22	AGK/SGK1
Congo	SGK	117	106	123	H22	AGK/SGK1
Congo	SGK	117	106	125	H22	AGK/SGK1
Congo	SGK	117	106	129	H22	AGK/SGK1
Congo	SGK	117	106	134	H22	AGK/SGK1
Congo	SGK	117	106	141	H22	AGK/SGK1
Congo	SGK	117	106	141	H22	AGK/SGK1
Congo	SGK	117	106	141	H22	AGK/SGK1
Congo	SGK	117	106	141	H22	AGK/SGK1
Congo	SGK	117	106	141	H22	AGK/SGK1
Congo	SGK	117	106	141	H22	AGK/SGK1
Congo	SGK	117	106	141	H22	AGK/SGK1
Congo	SGK	117	106	141	H22	AGK/SGK1
Congo	SGK	117	106	141	H22	AGK/SGK1
Congo	SGK	117	106	141	H22	AGK/SGK1
Congo	SGK	117	106	141	H22	AGK/SGK1
Congo	SGK	117	106	141	H22	AGK/SGK1
Congo	SGK	117	106	141	H22	AGK/SGK1
Congo	SGK	117	106	141	H22	AGK/SGK1
Congo	SGK	117	106	143	H22	AGK/SGK1
Congo	SGK	117	106	143	H22	AGK/SGK1
Congo	SGK	117	106	143	H22	AGK/SGK1
Congo	SGK	117	106	145	H22	AGK/SGK1
Congo	SGK	117	106	145	H22	AGK/SGK1
Congo	SGK	117	106	145	H22	AGK/SGK1
Congo	SGK	117	106	-	H22	AGK/SGK1
Congo	SGK	117	106	-	H22	AGK/SGK1
Congo	SGK	117	106	-	H22	AGK/SGK1

Population ID	DHPS	0.8kb	4.3kb	7.7kb	2 locus haplotype code	Lineage code
Congo	SGK	117	106	-	H22	AGK/SGK1
Congo	SGK	117	106	-	H22	AGK/SGK1
Congo	SGK	117	106	-	H22	AGK/SGK1
Congo	SGK	117	106	-	H22	AGK/SGK1
Congo	SGK	117	106	-	H22	AGK/SGK1
Congo	SGK	117	106	-	H22	AGK/SGK1
Congo	SGK	117	106	-	H22	AGK/SGK1
Congo	SGK	117	106	-	H22	AGK/SGK1
Congo	SGK	117	106	-	H22	AGK/SGK1
Congo	SGK	117	106	-	H22	AGK/SGK1
Congo	SGK	117	106	-	H22	AGK/SGK1
Congo	SGK	117	106	-	H22	AGK/SGK1
Congo	SGK	117	106	-	H22	AGK/SGK1
Congo	SGK	117	106	-	H22	AGK/SGK1
Congo	SGK	117	106	-	H22	AGK/SGK1
Congo	SGK	117	106	-	H22	AGK/SGK1
Congo	SGK	117	106	-	H22	AGK/SGK1
Congo	SGK	117	106	-	H22	AGK/SGK1
Congo	SGK	117	106	-	H22	AGK/SGK1
Congo	SGK	117	106	-	H22	AGK/SGK1
Congo	SGK	117	106	-	H22	AGK/SGK1
Congo	SGK	117	106	-	H22	AGK/SGK1
Congo	SGK	117	106	-	H22	AGK/SGK1
Congo	SGK	117	108	113	H23	AGK/SGK1
Congo	SGK	117	110	125	H24	AGK/SGK1
Congo	SGK	117	-	105		
Congo	SGK	117	-	113		
Congo	SGK	117	-	115		
Congo	SGK	117	-	115		
Congo	SGK	117	-	119		
Congo	SGK	117	-	123		
Congo	SGK	117	-	141		
Congo	SGK	117	-	-		
Congo	SGK	117	-	-		
Congo	SGK	119	110	123	H30	
Congo	SGK	121	106	115	H33	AGK/SGK2
Congo	SGK	121	106	117	H33	AGK/SGK2
Congo	SGK	121	108	123	H34	AGK/SGK2
Congo	SGK	121	110	119	H35	AGK/SGK2
Congo	SGK	123	-	119		
Congo	SGK	125	104	115	H50	
Congo	SGK	125	104	117	H50	
Congo	SGK	131	104	107	H66	

Population ID	DHPS	0.8kb	4.3kb	7.7kb	2 locus haplotype code	Lineage code
Congo	SGK	131	104	107	H66	
Congo	SGK	131	106	-	H67	
Congo	SGK	133	106	123	H77	
Congo	SGK	133	106	123	H77	
Congo	SGK	133	108	109	H78	
Congo	SGK	-	106	113		
Congo	SGK	-	106	119		
Congo	SGK	-	106	119		
Congo	SGK	-	106	141		
Congo	SGK	-	106	-		
Congo	SGK	-	108	-		
Congo	SGK	-	-	103		
Congo	SGK	-	-	107		
Congo	SGK	-	-	115		
Congo	SGK	-	-	129		
DRC	AAK	107	-	-		
DRC	AAK	113	104	-	H11	
DRC	AAK	113	106	129	H12	
DRC	AAK	113	106	-	H12	
DRC	AAK	113	106	-	H12	
DRC	AAK	113	-	127		
DRC	AAK	-	106	111		
DRC	AAK	-	106	119		
DRC	AAK	-	-	109		
DRC	SAK	113	112	123	H14	
DRC	SAK	115	-	105		
DRC	SAK	117	102	-	H20	
DRC	SAK	117	106	-	H22	
DRC	SAK	119	-	-		
DRC	SAK	125	104	113	H50	
DRC	SAK	125	104	117	H50	
DRC	SAK	125	108	125	H52	
DRC	SAK	125	108	125	H52	
DRC	SAK	125	110	111	H53	
DRC	SAK	127	-	117		
DRC	SAK	131	110	113	H69	
DRC	SAK	133	104	121	H76	
DRC	SAK	-	104	-		
DRC	SAK	-	104	-		
DRC	SAK	-	108	132		
DRC	SAK	-	110	115		
DRC	SAK	-	-	109		
DRC	SGE	131	100	105	H64	SGE1

Population ID	DHPS	0.8kb	4.3kb	7.7kb	2 locus haplotype code	Lineage code
DRC	SGE	131	104	105	H66	SGE1
DRC	SGE	131	104	107	H66	SGE1
DRC	SGE	131	104	107	H66	SGE1
DRC	SGE	131	104	107	H66	SGE1
DRC	SGE	131	104	107	H66	SGE1
DRC	SGE	131	104	107	H66	SGE1
DRC	SGE	131	104	107	H66	SGE1
DRC	SGE	131	104	107	H66	SGE1
DRC	SGE	131	104	107	H66	SGE1
DRC	SGE	131	104	107	H66	SGE1
DRC	SGE	131	104	107	H66	SGE1
DRC	SGE	131	104	107	H66	SGE1
DRC	SGE	131	104	107	H66	SGE1
DRC	SGE	131	104	107	H66	SGE1
DRC	SGE	131	104	107	H66	SGE1
DRC	SGE	131	104	107	H66	SGE1
DRC	SGE	131	104	107	H66	SGE1
DRC	SGE	131	104	107	H66	SGE1
DRC	SGE	131	104	107	H66	SGE1
DRC	SGE	131	104	107	H66	SGE1
DRC	SGE	131	104	107	H66	SGE1
DRC	SGE	131	104	107	H66	SGE1
DRC	SGE	131	104	107	H66	SGE1
DRC	SGE	131	104	107	H66	SGE1
DRC	SGE	131	104	107	H66	SGE1
DRC	SGE	131	104	107	H66	SGE1
DRC	SGE	131	104	107	H66	SGE1
DRC	SGE	131	104	107	H66	SGE1
DRC	SGE	131	104	117	H66	SGE1
DRC	SGE	131	104	-	H66	SGE1
DRC	SGE	131	108	119	H68	SGE1
DRC	SGE	133	104	-	H76	
DRC	SGE	136	104	107	H81	
DRC	SGE	-	104	107		
DRC	SGE	-	104	107		
DRC	SGE	-	104	107		
DRC	SGE	-	104	107		
DRC	SGE	-	104	107		
DRC	SGK	117	-	141		
Ethiopia	SAK	123	106	115	H43	
Ethiopia	SGE	121	114	98	H37	SGE2
Ethiopia	SGE	121	114	98	H37	SGE2
Ethiopia	SGE	121	114	98	H37	SGE2

Population ID	DHPS	0.8kb	4.3kb	7.7kb	2 locus haplotype code	Lineage code
Ethiopia	SGE	121	114	98	H37	SGE2
Ethiopia	SGE	121	114	98	H37	SGE2
Ethiopia	SGE	121	114	98	H37	SGE2
Ethiopia	SGE	121	114	98	H37	SGE2
Ethiopia	SGE	121	114	98	H37	SGE2
Ethiopia	SGE	121	114	98	H37	SGE2
Ethiopia	SGE	121	114	98	H37	SGE2
Ethiopia	SGE	121	114	98	H37	SGE2
Ethiopia	SGE	121	114	98	H37	SGE2
Ethiopia	SGE	121	114	98	H37	SGE2
Ethiopia	SGE	121	114	98	H37	SGE2
Ethiopia	SGE	121	114	98	H37	SGE2
Ethiopia	SGE	121	114	98	H37	SGE2
Ethiopia	SGE	121	114	98	H37	SGE2
Ethiopia	SGE	121	114	98	H37	SGE2
Ethiopia	SGE	121	114	98	H37	SGE2
Ethiopia	SGE	121	114	98	H37	SGE2
Ethiopia	SGE	121	114	98	H37	SGE2
Ethiopia	SGE	121	114	98	H37	SGE2
Ethiopia	SGE	121	114	98	H37	SGE2
Ethiopia	SGE	121	114	98	H37	SGE2
Ethiopia	SGE	121	114	98	H37	SGE2
Ethiopia	SGE	121	114	98	H37	SGE2
Ethiopia	SGE	121	114	98	H37	SGE2
Ethiopia	SGE	121	114	98	H37	SGE2
Ethiopia	SGE	121	114	98	H37	SGE2
Ethiopia	SGE	121	114	-	H37	SGE2
Ethiopia	SGE	123	114	98	H47	
Ethiopia	SGE	123	114	98	H47	
Ethiopia	SGE	131	104	107	H66	SGE1
Ethiopia	SGE	131	104	-	H66	SGE1
Ethiopia	SGE	131	106	119	H67	SGE1
Ethiopia	SGE	131	106	119	H67	SGE1
Ethiopia	SGK	121	114	98	H37	
Gabon	AAK	117	110	-	H24	
Gabon	AAK	121	110	119	H35	
Gabon	AAK	121	112	117	H36	
Gabon	AAK	121	-	117		
Gabon	AAK	121	-	117		
Gabon	AAK	125	106	117	H51	
Gabon	AAK	131	104	134	H66	
Gabon	AAK	131	118	125	H73	
Gabon	AAK	131	118	125	H73	

Population ID	DHPS	0.8kb	4.3kb	7.7kb	2 locus haplotype code	Lineage code
Gabon	AAK	131	118	125	H73	
Gabon	AAK	131	118	125	H73	
Gabon	AAK	131	118	127	H73	
Gabon	AAK	-	104	115		
Gabon	AAK	-	106	109		
Gabon	AAK	-	110	117		
Gabon	AGK	117	106	141	H22	AGK/SGK1
Gabon	AGK	121	108	113	H34	AGK/SGK2
Gabon	AGK	121	108	115	H34	AGK/SGK2
Gabon	AGK	121	110	111	H35	AGK/SGK2
Gabon	AGK	121	110	117	H35	AGK/SGK2
Gabon	AGK	121	110	-	H35	AGK/SGK2
Gabon	SAK	-	104	123		
Gabon	SGE	121	106	113	H33	
Gabon	SGE	131	104	107	H66	SGE1
Gabon	SGE	131	104	107	H66	SGE1
Gabon	SGE	131	104	125	H66	SGE1
Gabon	SGK	105	112	-	H03	
Gabon	SGK	105	-	141		
Gabon	SGK	117	104	123	H21	AGK/SGK1
Gabon	SGK	117	104	-	H21	AGK/SGK1
Gabon	SGK	117	106	119	H22	AGK/SGK1
Gabon	SGK	117	106	141	H22	AGK/SGK1
Gabon	SGK	117	106	141	H22	AGK/SGK1
Gabon	SGK	117	106	141	H22	AGK/SGK1
Gabon	SGK	117	106	141	H22	AGK/SGK1
Gabon	SGK	117	106	141	H22	AGK/SGK1
Gabon	SGK	117	106	141	H22	AGK/SGK1
Gabon	SGK	117	106	141	H22	AGK/SGK1
Gabon	SGK	117	106	141	H22	AGK/SGK1
Gabon	SGK	117	106	143	H22	AGK/SGK1
Gabon	SGK	117	106	-	H22	AGK/SGK1
Gabon	SGK	117	106	-	H22	AGK/SGK1
Gabon	SGK	117	106	-	H22	AGK/SGK1
Gabon	SGK	117	106	-	H22	AGK/SGK1
Gabon	SGK	117	106	-	H22	AGK/SGK1
Gabon	SGK	117	110	-	H24	AGK/SGK1
Gabon	SGK	117	112	-	H25	AGK/SGK1
Gabon	SGK	117	-	141		
Gabon	SGK	117	-	-		
Gabon	SGK	119	108	-	H29	
Gabon	SGK	119	116	141	H31	
Gabon	SGK	121	108	113	H34	AGK/SGK2

Population ID	DHPS	0.8kb	4.3kb	7.7kb	2 locus haplotype code	Lineage code
Gabon	SGK	121	116	113	H38	
Gabon	SGK	121	118	125	H39	
Gabon	SGK	123	108	109	H44	AGK/SGK3
Gabon	SGK	123	108	109	H44	AGK/SGK3
Gabon	SGK	125	108	109	H52	
Gabon	SGK	125	110	117	H53	
Gabon	SGK	131	108	-	H68	
Gabon	SGK	-	106	145		
Gabon	SGK	-	-	109		
Gabon	SGK	-	-	127		
Ghana	AAK	113	108	113	H13	
Ghana	AAK	115	106	113	H15	
Ghana	AAK	115	-	113		
Ghana	AAK	121	106	113	H33	
Ghana	AAK	121	108	113	H34	
Ghana	AAK	121	108	-	H34	
Ghana	AAK	-	104	121		
Ghana	AAK	-	106	113		
Ghana	AAK	-	106	113		
Ghana	AAK	-	108	113		
Ghana	AAK	-	-	113		
Ghana	AGK	103	108	113	H02	
Ghana	AGK	113	106	-	H12	
Ghana	AGK	115	106	113	H15	
Ghana	AGK	115	-	115		
Ghana	AGK	115	-	-		
Ghana	AGK	115	-	-		
Ghana	AGK	117	106	113	H22	AGK/SGK1
Ghana	AGK	119	-	-		
Ghana	AGK	121	108	113	H34	AGK/SGK2
Ghana	AGK	121	108	113	H34	AGK/SGK2
Ghana	AGK	121	108	113	H34	AGK/SGK2
Ghana	AGK	121	108	113	H34	AGK/SGK2
Ghana	AGK	121	108	113	H34	AGK/SGK2
Ghana	AGK	121	108	113	H34	AGK/SGK2
Ghana	AGK	121	108	113	H34	AGK/SGK2
Ghana	AGK	121	108	113	H34	AGK/SGK2
Ghana	AGK	121	108	113	H34	AGK/SGK2
Ghana	AGK	121	108	113	H34	AGK/SGK2
Ghana	AGK	121	108	113	H34	AGK/SGK2
Ghana	AGK	121	108	115	H34	AGK/SGK2
Ghana	AGK	121	108	-	H34	AGK/SGK2
Ghana	AGK	121	-	113		

Population ID	DHPS	0.8kb	4.3kb	7.7kb	2 locus haplotype code	Lineage code
Ghana	AGK	121	-	113		
Ghana	AGK	121	-	113		
Ghana	AGK	121	-	117		
Ghana	AGK	121	-	119		
Ghana	AGK	121	-	-		
Ghana	AGK	123	104	111	H42	AGK/SGK3
Ghana	AGK	123	108	113	H44	AGK/SGK3
Ghana	AGK	123	112	113	H46	
Ghana	AGK	125	104	-	H50	
Ghana	AGK	127	106	113	H55	
Ghana	AGK	133	104	111	H76	
Ghana	AGK	-	104	113		
Ghana	AGK	-	106	113		
Ghana	AGK	-	106	-		
Ghana	AGK	-	106	-		
Ghana	AGK	-	108	113		
Ghana	AGK	-	108	113		
Ghana	AGK	-	108	113		
Ghana	AGK	-	108	113		
Ghana	AGK	-	108	113		
Ghana	AGK	-	108	113		
Ghana	AGK	-	108	121		
Ghana	AGK	-	108	-		
Ghana	AGK	-	108	-		
Ghana	AGK	-	108	-		
Ghana	AGK	-	-	113		
Ghana	AGK	-	-	113		
Ghana	AGK	-	-	113		
Ghana	AGK	-	-	113		
Ghana	AGK	-	-	113		
Ghana	AGK	-	-	113		
Ghana	AGK	-	-	113		
Ghana	AGK	-	-	113		
Ghana	AGK	-	-	115		
Ghana	AGK	-	-	117		
Ghana	AGK	-	-	117		
Ghana	SGK	115	106	113	H15	
Ghana	SGK	115	106	-	H15	
Ghana	SGK	117	108	109	H23	AGK/SGK1
Ghana	SGK	121	108	113	H34	AGK/SGK2
Ghana	SGK	121	108	113	H34	AGK/SGK2
Ghana	SGK	121	108	113	H34	AGK/SGK2
Ghana	SGK	121	108	113	H34	AGK/SGK2

Population ID	DHPS	0.8kb	4.3kb	7.7kb	2 locus haplotype code	Lineage code
Ghana	SGK	121	108	113	H34	AGK/SGK2
Ghana	SGK	121	108	113	H34	AGK/SGK2
Ghana	SGK	121	108	113	H34	AGK/SGK2
Ghana	SGK	121	-	113		
Ghana	SGK	121	-	113		
Ghana	SGK	123	108	113	H44	AGK/SGK3
Ghana	SGK	127	106	-	H55	
Ghana	SGK	-	104	-		
Ghana	SGK	-	104	-		
Ghana	SGK	-	106	113		
Ghana	SGK	-	106	113		
Ghana	SGK	-	108	113		
Ghana	SGK	-	108	113		
Ghana	SGK	-	108	113		
Ghana	SGK	-	108	113		
Ghana	SGK	-	108	-		
Ghana	SGK	-	108	-		
Ghana	SGK	-	-	113		
Ghana	SGK	-	-	113		
Guinea	AAK	113	-	119		
Guinea	AAK	115	106	117	H15	
Guinea	AAK	115	106	121	H15	
Guinea	AAK	115	-	-		
Guinea	AAK	123	96	119	H49	
Guinea	AAK	123	106	113	H43	
Guinea	AAK	127	106	127	H55	
Guinea	AAK	127	106	-	H55	
Guinea	AAK	129	108	119	H61	
Guinea	AAK	129	108	119	H61	
Guinea	AAK	-	108	123		
Guinea	AAK	-	108	-		
Guinea	AGK	115	108	113	H16	
Guinea	AGK	115	108	113	H16	
Guinea	AGK	115	108	-	H16	
Guinea	AGK	115	-	-		
Guinea	AGK	121	104	119	H32	AGK/SGK2
Guinea	AGK	121	106	141	H33	AGK/SGK2
Guinea	AGK	121	108	105	H34	AGK/SGK2
Guinea	AGK	121	108	113	H34	AGK/SGK2
Guinea	AGK	121	108	113	H34	AGK/SGK2
Guinea	AGK	121	108	132	H34	AGK/SGK2
Guinea	AGK	121	-	113		
Guinea	AGK	121	-	113		

Population ID	DHPS	0.8kb	4.3kb	7.7kb	2 locus haplotype code	Lineage code
Guinea	AGK	121	-	113		
Guinea	AGK	121	-	113		
Guinea	AGK	123	108	113	H44	AGK/SGK3
Guinea	AGK	123	112	119	H46	
Guinea	AGK	127	106	113	H55	
Guinea	AGK	127	106	119	H55	
Guinea	SAK	121	-	113		
Guinea	SAK	127	104	117	H54	
Guinea	SAK	131	108	123	H68	
Guinea	SAK	133	104	117	H76	
Guinea	SAK	-	-	123		
Guinea	SGE	121	104	-	H32	
Guinea	SGE	131	104	113	H66	SGE1
Guinea	SGE	-	104	113		
Guinea	SGE	-	104	-		
Guinea	SGK	113	106	115	H12	
Guinea	SGK	117	106	-	H22	AGK/SGK1
Guinea	SGK	121	108	113	H34	AGK/SGK2
Guinea	SGK	121	108	113	H34	AGK/SGK2
Guinea	SGK	121	108	117	H34	AGK/SGK2
Guinea	SGK	121	108	117	H34	AGK/SGK2
Guinea	SGK	121	108	119	H34	AGK/SGK2
Guinea	SGK	121	118	117	H39	
Guinea	SGK	121	-	-		
Guinea	SGK	123	-	117		
Guinea	SGK	125	108	113	H52	
Guinea	SGK	129	106	113	H60	
Guinea	SGK	138	116	129	H84	
Guinea	SGK	141	116	-	H89	
Guinea	SGK	-	106	113		
Guinea	SGK	-	108	-		
Guinea	SGK	-	118	129		
Kenya	SGE	119	-	-		
Kenya	SGE	131	104	107	H66	SGE1
Kenya	SGE	131	104	107	H66	SGE1
Kenya	SGE	131	104	107	H66	SGE1
Kenya	SGE	131	104	107	H66	SGE1
Kenya	SGE	131	104	107	H66	SGE1
Kenya	SGE	131	104	107	H66	SGE1
Kenya	SGE	131	104	107	H66	SGE1
Kenya	SGE	131	104	107	H66	SGE1
Kenya	SGE	131	104	107	H66	SGE1
Kenya	SGE	131	104	107	H66	SGE1

Population ID	DHPS	0.8kb	4.3kb	7.7kb	2 locus haplotype code	Lineage code
Kenya	SGE	131	104	107	H66	SGE1
Kenya	SGE	131	104	107	H66	SGE1
Kenya	SGE	131	104	107	H66	SGE1
Kenya	SGE	131	104	107	H66	SGE1
Kenya	SGE	131	104	107	H66	SGE1
Kenya	SGE	131	104	107	H66	SGE1
Kenya	SGE	131	104	107	H66	SGE1
Kenya	SGE	131	104	107	H66	SGE1
Kenya	SGE	131	104	107	H66	SGE1
Kenya	SGE	131	104	107	H66	SGE1
Kenya	SGE	131	104	107	H66	SGE1
Kenya	SGE	131	104	107	H66	SGE1
Kenya	SGE	131	104	107	H66	SGE1
Kenya	SGE	131	104	107	H66	SGE1
Kenya	SGE	131	104	107	H66	SGE1
Kenya	SGE	131	104	107	H66	SGE1
Kenya	SGE	131	104	107	H66	SGE1
Kenya	SGE	131	104	107	H66	SGE1
Kenya	SGE	131	104	107	H66	SGE1
Kenya	SGE	131	104	107	H66	SGE1
Kenya	SGE	131	104	107	H66	SGE1
Kenya	SGE	131	104	107	H66	SGE1
Kenya	SGE	131	104	107	H66	SGE1
Kenya	SGE	131	104	107	H66	SGE1
Kenya	SGE	131	104	107	H66	SGE1
Kenya	SGE	131	104	107	H66	SGE1
Kenya	SGE	131	104	107	H66	SGE1
Kenya	SGE	131	104	107	H66	SGE1
Kenya	SGE	131	104	107	H66	SGE1
Kenya	SGE	131	104	107	H66	SGE1
Kenya	SGE	131	104	107	H66	SGE1
Kenya	SGE	131	104	107	H66	SGE1
Kenya	SGE	131	104	107	H66	SGE1
Kenya	SGE	131	104	107	H66	SGE1
Kenya	SGE	131	104	107	H66	SGE1
Kenya	SGE	131	104	107	H66	SGE1
Kenya	SGE	131	104	107	H66	SGE1
Kenya	SGE	131	104	107	H66	SGE1
Kenya	SGE	131	104	107	H66	SGE1
Kenya	SGE	131	104	107	H66	SGE1
Kenya	SGE	131	104	107	H66	SGE1
Kenya	SGE	131	104	107	H66	SGE1
Kenya	SGE	131	104	107	H66	SGE1

Population ID	DHPS	0.8kb	4.3kb	7.7kb	2 locus haplotype code	Lineage code
Kenya	SGE	131	104	107	H66	SGE1
Kenya	SGE	131	104	107	H66	SGE1
Kenya	SGE	131	104	107	H66	SGE1
Kenya	SGE	131	104	107	H66	SGE1
Kenya	SGE	131	104	107	H66	SGE1
Kenya	SGE	131	104	107	H66	SGE1
Kenya	SGE	131	104	107	H66	SGE1
Kenya	SGE	131	104	107	H66	SGE1
Kenya	SGE	131	104	109	H66	SGE1
Kenya	SGE	131	104	115	H66	SGE1
Kenya	SGE	131	104	119	H66	SGE1
Kenya	SGE	131	104	119	H66	SGE1
Kenya	SGE	131	104	-	H66	SGE1
Kenya	SGE	131	104	-	H66	SGE1
Kenya	SGE	131	104	-	H66	SGE1
Kenya	SGE	131	104	-	H66	SGE1
Kenya	SGE	131	104	-	H66	SGE1
Kenya	SGE	131	104	-	H66	SGE1
Kenya	SGE	131	104	-	H66	SGE1
Kenya	SGE	131	104	-	H66	SGE1
Kenya	SGE	131	104	-	H66	SGE1
Kenya	SGE	131	104	-	H66	SGE1
Kenya	SGE	131	104	-	H66	SGE1
Kenya	SGE	131	106	113	H67	SGE1
Kenya	SGE	131	114	-	H71	
Kenya	SGE	131	-	107		
Kenya	SGE	131	-	107		
Kenya	SGE	131	-	107		
Kenya	SGE	131	-	-		
Kenya	SGE	131	-	-		
Kenya	SGE	131	-	-		
Kenya	SGE	131	-	-		
Kenya	SGE	133	104	107	H76	
Kenya	SGE	141	104	107	H87	
Kenya	SGE	-	104	107		
Kenya	SGE	-	104	107		
Kenya	SGE	-	104	107		
Kenya	SGE	-	104	107		
Kenya	SGE	-	104	107		
Kenya	SGE	-	104	107		
Kenya	SGE	-	104	107		
Kenya	SGE	-	104	107		
Kenya	SGE	-	104	107		

Population ID	DHPS	0.8kb	4.3kb	7.7kb	2 locus haplotype code	Lineage code
Kenya	SGE	-	104	107		
Kenya	SGE	-	104	107		
Kenya	SGE	-	104	107		
Kenya	SGE	-	104	107		
Kenya	SGE	-	104	109		
Kenya	SGE	-	104	121		
Kenya	SGE	-	104	-		
Kenya	SGE	-	104	-		
Kenya	SGE	-	104	-		
Kenya	SGE	-	106	-		
Kenya	SGE	-	-	107		
Kenya	SGE	-	-	113		
Kenya	SGK	119	104	121	H27	
Kenya	SGK	119	104	-	H27	
Mozambique	AAK	119	-	111		
Mozambique	AAK	119	-	-		
Mozambique	AAK	131	104	-	H66	
Mozambique	AAK	-	104	-		
Mozambique	AAK	-	106	-		
Mozambique	AAK	-	-	107		
Mozambique	SAK	105	-	-		
Mozambique	SAK	111	-	111		
Mozambique	SAK	113	104	111	H11	
Mozambique	SAK	113	-	-		
Mozambique	SAK	115	104	117	H15	
Mozambique	SAK	115	110	111	H17	
Mozambique	SAK	115	110	117	H17	
Mozambique	SAK	115	112	117	H18	
Mozambique	SAK	117	104	117	H21	
Mozambique	SAK	117	104	121	H21	
Mozambique	SAK	117	104	-	H21	
Mozambique	SAK	117	106	-	H22	
Mozambique	SAK	117	-	-		
Mozambique	SAK	119	106	111	H28	
Mozambique	SAK	119	108	111	H29	
Mozambique	SAK	119	108	-	H29	
Mozambique	SAK	119	-	111		
Mozambique	SAK	119	-	-		
Mozambique	SAK	119	-	-		
Mozambique	SAK	119	-	-		
Mozambique	SAK	119	-	-		
Mozambique	SAK	119	-	-		
Mozambique	SAK	121	104	111	H32	

Population ID	DHPS	0.8kb	4.3kb	7.7kb	2 locus haplotype code	Lineage code
Mozambique	SAK	121	106	113	H33	
Mozambique	SAK	121	106	-	H33	
Mozambique	SAK	121	106	-	H33	
Mozambique	SAK	121	110	119	H35	
Mozambique	SAK	121	110	-	H35	
Mozambique	SAK	121	-	117		
Mozambique	SAK	121	-	129		
Mozambique	SAK	121	-	-		
Mozambique	SAK	121	-	-		
Mozambique	SAK	121	-	-		
Mozambique	SAK	121	-	-		
Mozambique	SAK	123	110	-	H45	
Mozambique	SAK	125	-	-		
Mozambique	SAK	127	104	119	H54	
Mozambique	SAK	127	104	-	H54	
Mozambique	SAK	129	106	113	H60	
Mozambique	SAK	129	106	127	H60	
Mozambique	SAK	129	-	-		
Mozambique	SAK	131	112	115	H70	
Mozambique	SAK	133	104	127	H76	
Mozambique	SAK	136	112	117	H83	
Mozambique	SAK	-	104	109		
Mozambique	SAK	-	104	117		
Mozambique	SAK	-	104	119		
Mozambique	SAK	-	104	119		
Mozambique	SAK	-	104	132		
Mozambique	SAK	-	104	-		
Mozambique	SAK	-	104	-		
Mozambique	SAK	-	106	113		
Mozambique	SAK	-	106	113		
Mozambique	SAK	-	106	115		
Mozambique	SAK	-	106	127		
Mozambique	SAK	-	106	-		
Mozambique	SAK	-	106	-		
Mozambique	SAK	-	106	-		
Mozambique	SAK	-	106	-		
Mozambique	SAK	-	106	-		
Mozambique	SAK	-	106	-		
Mozambique	SAK	-	106	-		
Mozambique	SAK	-	106	-		
Mozambique	SAK	-	108	115		
Mozambique	SAK	-	108	121		
Mozambique	SAK	-	110	115		

Population ID	DHPS	0.8kb	4.3kb	7.7kb	2 locus haplotype code	Lineage code
Mozambique	SAK	-	110	119		
Mozambique	SAK	-	-	111		
Mozambique	SAK	-	-	113		
Mozambique	SAK	-	-	115		
Mozambique	SAK	-	-	115		
Mozambique	SAK	-	-	117		
Mozambique	SGE	115	104	-	H15	
Mozambique	SGE	131	104	107	H66	SGE1
Mozambique	SGE	131	104	107	H66	SGE1
Mozambique	SGE	131	104	107	H66	SGE1
Mozambique	SGE	131	104	107	H66	SGE1
Mozambique	SGE	131	104	107	H66	SGE1
Mozambique	SGE	131	104	107	H66	SGE1
Mozambique	SGE	131	104	107	H66	SGE1
Mozambique	SGE	131	104	107	H66	SGE1
Mozambique	SGE	131	104	107	H66	SGE1
Mozambique	SGE	131	104	107	H66	SGE1
Mozambique	SGE	131	104	111	H66	SGE1
Mozambique	SGE	131	104	-	H66	SGE1
Mozambique	SGE	131	104	-	H66	SGE1
Mozambique	SGE	131	104	-	H66	SGE1
Mozambique	SGE	131	104	-	H66	SGE1
Mozambique	SGE	131	106	-	H67	SGE1
Mozambique	SGE	131	-	123		
Mozambique	SGE	-	104	107		
Mozambique	SGE	-	104	107		
Mozambique	SGE	-	104	107		
Mozambique	SGE	-	104	107		
Mozambique	SGE	-	104	107		
Mozambique	SGE	-	104	107		
Mozambique	SGE	-	104	107		
Mozambique	SGE	-	104	107		
Mozambique	SGE	-	104	107		
Mozambique	SGE	-	104	-		
Mozambique	SGE	-	104	-		
Mozambique	SGE	-	-	123		
Mozambique	SGK	121	-	119		
Mozambique	SGK	-	106	-		
Namibia	AGK	117	106	127	H22	AGK/SGK1
Namibia	AGK	117	106	127	H22	AGK/SGK1
Namibia	AGK	117	106	141	H22	AGK/SGK1
Namibia	AGK	117	106	141	H22	AGK/SGK1
Namibia	AGK	117	106	-	H22	AGK/SGK1

Population ID	DHPS	0.8kb	4.3kb	7.7kb	2 locus haplotype code	Lineage code
Namibia	AGK	117	106	-	H22	AGK/SGK1
Namibia	AGK	117	-	117		
Namibia	AGK	117	-	125		
Namibia	AGK	-	110	-		
Namibia	SAK	117	108	117	H23	
Namibia	SAK	117	110	-	H24	
Namibia	SAK	119	110	117	H30	
Namibia	SAK	119	-	-		
Namibia	SAK	121	106	129	H33	
Namibia	SAK	123	104	121	H42	
Namibia	SAK	123	104	-	H42	
Namibia	SAK	127	104	123	H54	
Namibia	SAK	131	104	123	H66	
Namibia	SAK	-	106	113		
Namibia	SGE	131	104	107	H66	SGE1
Namibia	SGE	131	104	107	H66	SGE1
Namibia	SGE	131	104	107	H66	SGE1
Namibia	SGE	131	110	115	H69	SGE1
Namibia	SGE	-	104	107		
Namibia	SGE	-	106	-		
Namibia	SGE	-	-	107		
Namibia	SGK	117	104	107	H21	AGK/SGK1
Namibia	SGK	117	106	119	H22	AGK/SGK1
Namibia	SGK	117	106	121	H22	AGK/SGK1
Namibia	SGK	117	106	125	H22	AGK/SGK1
Namibia	SGK	117	106	127	H22	AGK/SGK1
Namibia	SGK	117	106	127	H22	AGK/SGK1
Namibia	SGK	117	106	141	H22	AGK/SGK1
Namibia	SGK	117	106	141	H22	AGK/SGK1
Namibia	SGK	117	106	141	H22	AGK/SGK1
Namibia	SGK	117	106	141	H22	AGK/SGK1
Namibia	SGK	117	106	141	H22	AGK/SGK1
Namibia	SGK	117	106	141	H22	AGK/SGK1
Namibia	SGK	117	106	141	H22	AGK/SGK1
Namibia	SGK	117	106	141	H22	AGK/SGK1
Namibia	SGK	117	106	141	H22	AGK/SGK1
Namibia	SGK	117	106	141	H22	AGK/SGK1
Namibia	SGK	117	106	141	H22	AGK/SGK1
Namibia	SGK	117	106	143	H22	AGK/SGK1
Namibia	SGK	117	106	143	H22	AGK/SGK1
Namibia	SGK	117	106	-	H22	AGK/SGK1
Namibia	SGK	117	106	-	H22	AGK/SGK1
Namibia	SGK	117	106	-	H22	AGK/SGK1

Population ID	DHPS	0.8kb	4.3kb	7.7kb	2 locus haplotype code	Lineage code
Namibia	SGK	117	106	-	H22	AGK/SGK1
Namibia	SGK	117	106	-	H22	AGK/SGK1
Namibia	SGK	117	106	-	H22	AGK/SGK1
Namibia	SGK	117	106	-	H22	AGK/SGK1
Namibia	SGK	117	106	-	H22	AGK/SGK1
Namibia	SGK	117	106	-	H22	AGK/SGK1
Namibia	SGK	117	106	-	H22	AGK/SGK1
Namibia	SGK	117	106	-	H22	AGK/SGK1
Namibia	SGK	117	106	-	H22	AGK/SGK1
Namibia	SGK	117	106	-	H22	AGK/SGK1
Namibia	SGK	117	106	-	H22	AGK/SGK1
Namibia	SGK	117	108	107	H23	AGK/SGK1
Namibia	SGK	117	108	117	H23	AGK/SGK1
Namibia	SGK	117	108	141	H23	AGK/SGK1
Namibia	SGK	117	108	-	H23	AGK/SGK1
Namibia	SGK	117	108	-	H23	AGK/SGK1
Namibia	SGK	117	108	-	H23	AGK/SGK1
Namibia	SGK	117	-	127		
Namibia	SGK	117	-	-		
Namibia	SGK	117	-	-		
Namibia	SGK	117	-	-		
Namibia	SGK	117	-	-		
Namibia	SGK	119	106	-	H28	
Namibia	SGK	119	-	141		
Namibia	SGK	119	-	-		
Namibia	SGK	125	110	121	H53	
Namibia	SGK	129	104	139	H59	
Nigeria	AAK	131	112	117	H70	
Nigeria	AAK	131	118	125	H73	
Nigeria	AAK	131	118	125	H73	
Nigeria	AAK	133	102	-	H75	
Nigeria	AAK	-	104	119		
Nigeria	AGK	121	110	111	H35	AGK/SGK2
Nigeria	AGK	121	110	117	H35	AGK/SGK2
Nigeria	SAK	117	108	123	H23	
Nigeria	SAK	121	110	-	H35	
Nigeria	SAK	129	114	107	H62	
Nigeria	SGK	121	108	113	H34	AGK/SGK2
Nigeria	SGK	121	108	-	H34	AGK/SGK2
Nigeria	SGK	123	108	109	H44	AGK/SGK3
Nigeria	SGK	-	116	-		
Nigeria	SGK	-	-	119		
Senegal	AAK	121	104	117	H32	

Population ID	DHPS	0.8kb	4.3kb	7.7kb	2 locus haplotype code	Lineage code
Senegal	AAK	127	104	115	H54	
Senegal	AAK	127	104	123	H54	
Senegal	AAK	127	108	119	H56	
Senegal	AAK	129	104	-	H59	
Senegal	AAK	129	108	119	H61	
Senegal	AAK	129	108	119	H61	
Senegal	AAK	129	108	119	H61	
Senegal	AAK	-	108	119		
Senegal	AGK	121	108	113	H34	AGK/SGK2
Senegal	SAK	111	108	119	H09	
Senegal	SAK	117	104	111	H21	
Senegal	SAK	121	106	125	H33	
Senegal	SAK	125	110	105	H53	
Senegal	SAK	127	108	115	H56	
Senegal	SAK	127	112	111	H57	
Senegal	SAK	129	106	115	H60	
Senegal	SAK	133	104	117	H76	
Senegal	SAK	-	104	-		
Senegal	SGK	117	106	141	H22	AGK/SGK1
Senegal	SGK	117	106	141	H22	AGK/SGK1
Senegal	SGK	117	106	141	H22	AGK/SGK1
Senegal	SGK	117	106	143	H22	AGK/SGK1
Senegal	SGK	117	106	143	H22	AGK/SGK1
Senegal	SGK	117	106	-	H22	AGK/SGK1
Senegal	SGK	117	106	-	H22	AGK/SGK1
Senegal	SGK	117	108	109	H23	AGK/SGK1
Senegal	SGK	117	116	141	H26	AGK/SGK1
Senegal	SGK	117	-	-		
Senegal	SGK	121	108	113	H34	AGK/SGK2
Senegal	SGK	121	108	113	H34	AGK/SGK2
Senegal	SGK	121	108	113	H34	AGK/SGK2
Senegal	SGK	121	108	113	H34	AGK/SGK2
Senegal	SGK	121	108	113	H34	AGK/SGK2
Senegal	SGK	121	108	113	H34	AGK/SGK2
Senegal	SGK	121	108	113	H34	AGK/SGK2
Senegal	SGK	121	108	113	H34	AGK/SGK2
Senegal	SGK	121	108	119	H34	AGK/SGK2
Senegal	SGK	121	108	119	H34	AGK/SGK2
Senegal	SGK	121	112	105	H36	AGK/SGK2
Senegal	SGK	121	-	113		
Senegal	SGK	123	108	109	H44	AGK/SGK3
Senegal	SGK	-	106	-		
Senegal	SGK	-	-	117		

Population ID	DHPS	0.8kb	4.3kb	7.7kb	2 locus haplotype code	Lineage code
South Africa	SGE	131	104	107	H66	SGE1
South Africa	SGE	131	104	107	H66	SGE1
South Africa	SGE	131	104	107	H66	SGE1
South Africa	SGE	131	104	107	H66	SGE1
South Africa	SGE	131	104	107	H66	SGE1
South Africa	SGE	131	104	107	H66	SGE1
South Africa	SGE	131	104	107	H66	SGE1
South Africa	SGE	131	104	107	H66	SGE1
South Africa	SGE	131	104	107	H66	SGE1
South Africa	SGE	131	104	107	H66	SGE1
South Africa	SGE	131	104	107	H66	SGE1
South Africa	SGE	131	104	107	H66	SGE1
South Africa	SGE	131	104	107	H66	SGE1
South Africa	SGE	131	104	107	H66	SGE1
South Africa	SGE	131	104	107	H66	SGE1
South Africa	SGE	131	104	107	H66	SGE1
South Africa	SGE	131	104	107	H66	SGE1
South Africa	SGE	131	104	107	H66	SGE1
South Africa	SGE	131	104	107	H66	SGE1
South Africa	SGE	131	104	107	H66	SGE1
South Africa	SGE	131	104	107	H66	SGE1
South Africa	SGE	131	104	107	H66	SGE1
South Africa	SGE	131	104	107	H66	SGE1
South Africa	SGE	131	104	107	H66	SGE1
South Africa	SGE	131	104	109	H66	SGE1
South Africa	SGE	131	104	109	H66	SGE1
South Africa	SGE	131	104	115	H66	SGE1
Sudan	SGE	121	110	-	H35	
Sudan	SGE	121	114	98	H37	SGE2
Sudan	SGE	121	114	98	H37	SGE2
Sudan	SGE	121	114	98	H37	SGE2
Sudan	SGE	121	114	98	H37	SGE2
Sudan	SGE	121	114	98	H37	SGE2
Sudan	SGE	121	114	98	H37	SGE2
Sudan	SGE	121	114	98	H37	SGE2
Sudan	SGE	121	114	98	H37	SGE2
Sudan	SGE	121	114	98	H37	SGE2
Sudan	SGE	121	114	98	H37	SGE2
Sudan	SGE	121	114	98	H37	SGE2
Sudan	SGE	121	114	98	H37	SGE2
Sudan	SGE	121	114	98	H37	SGE2
Sudan	SGE	121	114	98	H37	SGE2
Sudan	SGE	121	114	98	H37	SGE2

Population ID	DHPS	0.8kb	4.3kb	7.7kb	2 locus haplotype code	Lineage code
Sudan	SGE	121	114	98	H37	SGE2
Sudan	SGE	121	114	98	H37	SGE2
Sudan	SGE	121	114	98	H37	SGE2
Sudan	SGE	121	114	98	H37	SGE2
Sudan	SGE	121	114	98	H37	SGE2
Sudan	SGE	121	114	98	H37	SGE2
Sudan	SGE	121	114	98	H37	SGE2
Sudan	SGE	121	114	98	H37	SGE2
Sudan	SGE	121	114	98	H37	SGE2
Sudan	SGE	121	114	98	H37	SGE2
Sudan	SGE	121	114	98	H37	SGE2
Sudan	SGE	121	114	98	H37	SGE2
Sudan	SGE	121	114	98	H37	SGE2
Sudan	SGE	121	114	98	H37	SGE2
Sudan	SGE	121	114	98	H37	SGE2
Sudan	SGE	121	114	98	H37	SGE2
Sudan	SGE	121	114	98	H37	SGE2
Sudan	SGE	121	114	98	H37	SGE2
Sudan	SGE	121	114	98	H37	SGE2
Sudan	SGE	121	114	98	H37	SGE2
Sudan	SGE	121	114	98	H37	SGE2
Sudan	SGE	121	114	98	H37	SGE2
Sudan	SGE	121	114	98	H37	SGE2
Sudan	SGE	121	114	98	H37	SGE2
Sudan	SGE	121	114	98	H37	SGE2
Sudan	SGE	121	114	98	H37	SGE2
Sudan	SGE	121	114	98	H37	SGE2
Sudan	SGE	121	114	98	H37	SGE2
Sudan	SGE	121	114	98	H37	SGE2
Sudan	SGE	121	114	98	H37	SGE2
Sudan	SGE	121	114	98	H37	SGE2
Sudan	SGE	121	114	98	H37	SGE2
Sudan	SGE	121	114	98	H37	SGE2
Sudan	SGE	121	114	98	H37	SGE2
Sudan	SGE	121	114	98	H37	SGE2
Sudan	SGE	121	114	98	H37	SGE2
Sudan	SGE	121	114	98	H37	SGE2
Sudan	SGE	121	114	98	H37	SGE2
Sudan	SGE	121	114	98	H37	SGE2
Sudan	SGE	121	114	98	H37	SGE2
Sudan	SGE	121	114	98	H37	SGE2
Sudan	SGE	121	114	121	H37	SGE2
Sudan	SGE	121	114	-	H37	SGE2

Population ID	DHPS	0.8kb	4.3kb	7.7kb	2 locus haplotype code	Lineage code
Sudan	SGE	121	-	98		
Sudan	SGE	121	-	98		
Sudan	SGE	121	-	98		
Sudan	SGE	121	-	98		
Sudan	SGE	121	-	-		
Sudan	SGE	121	-	-		
Sudan	SGE	-	114	98		
Sudan	SGE	-	114	-		
Sudan	SGK	121	114	-	H37	
Tanzania	AAK	115	106	125	H15	
Tanzania	AAK	119	-	107		
Tanzania	AAK	125	106	-	H51	
Tanzania	AAK	127	-	-		
Tanzania	AAK	136	104	127	H81	
Tanzania	AAK	136	104	143	H81	
Tanzania	AAK	136	106	-	H82	
Tanzania	AAK	-	104	111		
Tanzania	AAK	-	104	-		
Tanzania	AAK	-	106	117		
Tanzania	AAK	-	-	107		
Tanzania	SAK	115	-	117		
Tanzania	SAK	117	104	-	H21	
Tanzania	SAK	117	-	-		
Tanzania	SAK	121	94	123	H40	
Tanzania	SAK	125	108	113	H52	
Tanzania	SAK	127	104	107	H54	
Tanzania	SAK	127	-	113		
Tanzania	SAK	129	-	111		
Tanzania	SAK	131	104	117	H66	
Tanzania	SAK	131	104	-	H66	
Tanzania	SAK	131	112	115	H70	
Tanzania	SAK	131	-	-		
Tanzania	SAK	133	108	-	H78	
Tanzania	SAK	140	104	107	H85	
Tanzania	SAK	140	108	119	H86	
Tanzania	SAK	-	104	-		
Tanzania	SAK	-	104	-		
Tanzania	SAK	-	106	-		
Tanzania	SAK	-	106	-		
Tanzania	SAK	-	106	-		
Tanzania	SAK	-	110	103		
Tanzania	SAK	-	110	109		
Tanzania	SAK	-	118	117		

Population ID	DHPS	0.8kb	4.3kb	7.7kb	2 locus haplotype code	Lineage code
Tanzania	SAK	-	-	103		
Tanzania	SAK	-	-	107		
Tanzania	SGE	123	104	-	H42	
Tanzania	SGE	123	-	107		
Tanzania	SGE	125	106	117	H51	
Tanzania	SGE	129	104	101	H59	
Tanzania	SGE	131	104	107	H66	SGE1
Tanzania	SGE	131	104	107	H66	SGE1
Tanzania	SGE	131	104	107	H66	SGE1
Tanzania	SGE	131	104	107	H66	SGE1
Tanzania	SGE	131	104	107	H66	SGE1
Tanzania	SGE	131	104	107	H66	SGE1
Tanzania	SGE	131	104	107	H66	SGE1
Tanzania	SGE	131	104	107	H66	SGE1
Tanzania	SGE	131	104	107	H66	SGE1
Tanzania	SGE	131	104	107	H66	SGE1
Tanzania	SGE	131	104	107	H66	SGE1
Tanzania	SGE	131	104	107	H66	SGE1
Tanzania	SGE	131	104	107	H66	SGE1
Tanzania	SGE	131	104	107	H66	SGE1
Tanzania	SGE	131	104	107	H66	SGE1
Tanzania	SGE	131	104	107	H66	SGE1
Tanzania	SGE	131	104	107	H66	SGE1
Tanzania	SGE	131	104	107	H66	SGE1
Tanzania	SGE	131	104	107	H66	SGE1
Tanzania	SGE	131	104	107	H66	SGE1
Tanzania	SGE	131	104	107	H66	SGE1
Tanzania	SGE	131	104	107	H66	SGE1
Tanzania	SGE	131	104	119	H66	SGE1
Tanzania	SGE	131	104	125	H66	SGE1
Tanzania	SGE	131	104	-	H66	SGE1
Tanzania	SGE	131	110	113	H69	SGE1
Tanzania	SGE	131	110	125	H69	SGE1
Tanzania	SGE	131	-	115		
Tanzania	SGE	131	-	-		
Tanzania	SGE	133	104	107	H76	
Tanzania	SGE	133	104	107	H76	
Tanzania	SGE	133	104	107	H76	
Tanzania	SGE	-	104	107		
Tanzania	SGE	-	104	107		
Tanzania	SGE	-	104	129		
Tanzania	SGE	-	104	-		
Tanzania	SGE	-	104	-		

Population ID	DHPS	0.8kb	4.3kb	7.7kb	2 locus haplotype code	Lineage code
Tanzania	SGE	-	104	-		
Tanzania	SGE	-	104	-		
Tanzania	SGE	-	104	-		
Tanzania	SGE	-	104	-		
Tanzania	SGE	-	106	-		
Tanzania	SGE	-	-	107		
Tanzania	SGE	-	-	107		
Tanzania	SGE	-	-	107		
Tanzania	SGE	-	-	113		
Tanzania	SGK	119	108	-	H29	
Tanzania	SGK	131	-	-		
Tanzania	SGK	-	-	113		
Uganda	SAK	131	106	109	H67	
Uganda	SGE	103	104	-	H01	
Uganda	SGE	123	108	109	H44	
Uganda	SGE	123	108	109	H44	
Uganda	SGE	125	104	-	H50	
Uganda	SGE	131	96	-	H74	
Uganda	SGE	131	102	-	H65	SGE1
Uganda	SGE	131	102	-	H65	
Uganda	SGE	131	104	107	H66	SGE1
Uganda	SGE	131	104	107	H66	SGE1
Uganda	SGE	131	104	107	H66	SGE1
Uganda	SGE	131	104	107	H66	SGE1
Uganda	SGE	131	104	107	H66	SGE1
Uganda	SGE	131	104	107	H66	SGE1
Uganda	SGE	131	104	107	H66	SGE1
Uganda	SGE	131	104	107	H66	SGE1
Uganda	SGE	131	104	107	H66	SGE1
Uganda	SGE	131	104	107	H66	SGE1
Uganda	SGE	131	104	107	H66	SGE1
Uganda	SGE	131	104	107	H66	SGE1
Uganda	SGE	131	104	107	H66	SGE1
Uganda	SGE	131	104	107	H66	SGE1
Uganda	SGE	131	104	107	H66	SGE1
Uganda	SGE	131	104	107	H66	SGE1
Uganda	SGE	131	104	107	H66	SGE1
Uganda	SGE	131	104	107	H66	SGE1
Uganda	SGE	131	104	107	H66	SGE1
Uganda	SGE	131	104	107	H66	SGE1
Uganda	SGE	131	104	107	H66	SGE1
Uganda	SGE	131	104	107	H66	SGE1
Uganda	SGE	131	104	107	H66	SGE1

Population ID	DHPS	0.8kb	4.3kb	7.7kb	2 locus haplotype code	Lineage code
Uganda	SGE	131	104	107	H66	SGE1
Uganda	SGE	131	104	107	H66	SGE1
Uganda	SGE	131	104	107	H66	SGE1
Uganda	SGE	131	104	107	H66	SGE1
Uganda	SGE	131	104	107	H66	SGE1
Uganda	SGE	131	104	107	H66	SGE1
Uganda	SGE	131	104	107	H66	SGE1
Uganda	SGE	131	104	107	H66	SGE1
Uganda	SGE	131	104	107	H66	SGE1
Uganda	SGE	131	104	107	H66	SGE1
Uganda	SGE	131	104	107	H66	SGE1
Uganda	SGE	131	104	107	H66	SGE1
Uganda	SGE	131	104	107	H66	SGE1
Uganda	SGE	131	104	107	H66	SGE1
Uganda	SGE	131	104	107	H66	SGE1
Uganda	SGE	131	104	107	H66	SGE1
Uganda	SGE	131	104	107	H66	SGE1
Uganda	SGE	131	104	107	H66	SGE1
Uganda	SGE	131	104	107	H66	SGE1
Uganda	SGE	131	104	107	H66	SGE1
Uganda	SGE	131	104	107	H66	SGE1
Uganda	SGE	131	104	107	H66	SGE1
Uganda	SGE	131	104	107	H66	SGE1
Uganda	SGE	131	104	107	H66	SGE1
Uganda	SGE	131	104	107	H66	SGE1
Uganda	SGE	131	104	107	H66	SGE1
Uganda	SGE	131	104	107	H66	SGE1
Uganda	SGE	131	104	107	H66	SGE1
Uganda	SGE	131	104	107	H66	SGE1
Uganda	SGE	131	104	107	H66	SGE1
Uganda	SGE	131	104	107	H66	SGE1
Uganda	SGE	131	104	107	H66	SGE1
Uganda	SGE	131	104	107	H66	SGE1
Uganda	SGE	131	104	107	H66	SGE1
Uganda	SGE	131	104	107	H66	SGE1
Uganda	SGE	131	104	107	H66	SGE1
Uganda	SGE	131	104	107	H66	SGE1
Uganda	SGE	131	104	107	H66	SGE1
Uganda	SGE	131	104	107	H66	SGE1
Uganda	SGE	131	104	107	H66	SGE1
Uganda	SGE	131	104	107	H66	SGE1
Uganda	SGE	131	104	107	H66	SGE1
Uganda	SGE	131	104	107	H66	SGE1

Population ID	DHPS	0.8kb	4.3kb	7.7kb	2 locus haplotype code	Lineage code
Uganda	SGE	131	104	107	H66	SGE1
Uganda	SGE	131	104	107	H66	SGE1
Uganda	SGE	131	104	107	H66	SGE1
Uganda	SGE	131	104	107	H66	SGE1
Uganda	SGE	131	104	107	H66	SGE1
Uganda	SGE	131	104	107	H66	SGE1
Uganda	SGE	131	104	107	H66	SGE1
Uganda	SGE	131	104	107	H66	SGE1
Uganda	SGE	131	104	107	H66	SGE1
Uganda	SGE	131	104	107	H66	SGE1
Uganda	SGE	131	104	107	H66	SGE1
Uganda	SGE	131	104	107	H66	SGE1
Uganda	SGE	131	104	107	H66	SGE1
Uganda	SGE	131	104	107	H66	SGE1
Uganda	SGE	131	104	107	H66	SGE1
Uganda	SGE	131	104	109	H66	SGE1
Uganda	SGE	131	104	111	H66	SGE1
Uganda	SGE	131	104	113	H66	SGE1
Uganda	SGE	131	104	113	H66	SGE1
Uganda	SGE	131	104	113	H66	SGE1
Uganda	SGE	131	104	117	H66	SGE1
Uganda	SGE	131	104	125	H66	SGE1
Uganda	SGE	131	104	125	H66	SGE1
Uganda	SGE	131	104	-	H66	SGE1
Uganda	SGE	131	104	-	H66	SGE1
Uganda	SGE	131	104	-	H66	SGE1
Uganda	SGE	131	104	-	H66	SGE1
Uganda	SGE	131	104	-	H66	SGE1
Uganda	SGE	131	104	-	H66	SGE1
Uganda	SGE	131	104	-	H66	SGE1
Uganda	SGE	131	104	-	H66	SGE1
Uganda	SGE	131	104	-	H66	SGE1
Uganda	SGE	131	104	-	H66	SGE1
Uganda	SGE	131	104	-	H66	SGE1
Uganda	SGE	131	104	-	H66	SGE1
Uganda	SGE	131	104	-	H66	SGE1
Uganda	SGE	131	104	-	H66	SGE1
Uganda	SGE	131	104	-	H66	SGE1
Uganda	SGE	131	104	-	H66	SGE1
Uganda	SGE	131	104	-	H66	SGE1
Uganda	SGE	131	104	-	H66	SGE1
Uganda	SGE	131	110	-	H69	SGE1
Uganda	SGE	131	-	-		
Uganda	SGE	131	-	-		

Population ID	DHPS	0.8kb	4.3kb	7.7kb	2 locus haplotype code	Lineage code
Uganda	SGE	133	104	107	H76	
Uganda	SGE	133	104	136	H76	
Uganda	SGE	136	104	107	H81	
Uganda	SGE	136	104	107	H81	
Uganda	SGE	-	104	107		
Uganda	SGE	-	104	107		
Uganda	SGE	-	104	107		
Uganda	SGE	-	104	107		
Uganda	SGE	-	104	107		
Uganda	SGE	-	104	107		
Uganda	SGE	-	104	-		
Uganda	SGE	-	104	-		
Zambia	AAK	136	104	127	H81	
Zambia	AAK	-	104	117		
Zambia	SAK	113	108	115	H13	
Zambia	SAK	115	106	105	H15	
Zambia	SAK	117	104	117	H21	
Zambia	SAK	117	106	-	H22	
Zambia	SAK	117	110	-	H24	
Zambia	SAK	117	-	-		
Zambia	SAK	119	104	107	H27	
Zambia	SAK	119	104	113	H27	
Zambia	SAK	119	106	111	H28	
Zambia	SAK	119	-	127		
Zambia	SAK	119	-	-		
Zambia	SAK	119	-	-		
Zambia	SAK	121	98	123	H41	
Zambia	SAK	121	104	115	H32	
Zambia	SAK	123	108	119	H44	
Zambia	SAK	123	-	121		
Zambia	SAK	125	108	-	H52	
Zambia	SAK	127	106	107	H55	
Zambia	SAK	127	114	123	H58	
Zambia	SAK	127	-	-		
Zambia	SAK	129	106	113	H60	
Zambia	SAK	129	108	-	H61	
Zambia	SAK	131	104	117	H66	
Zambia	SAK	131	106	123	H67	
Zambia	SAK	133	104	115	H76	
Zambia	SAK	133	104	115	H76	
Zambia	SAK	133	106	105	H77	
Zambia	SAK	133	106	-	H77	
Zambia	SAK	-	100	121		

Population ID	DHPS	0.8kb	4.3kb	7.7kb	2 locus haplotype code	Lineage code
Zambia	SAK	-	104	119		
Zambia	SAK	-	104	121		
Zambia	SAK	-	106	115		
Zambia	SAK	-	106	115		
Zambia	SAK	-	106	-		
Zambia	SAK	-	106	-		
Zambia	SAK	-	108	129		
Zambia	SAK	-	-	111		
Zambia	SAK	-	-	113		
Zambia	SGE	117	106	107	H22	
Zambia	SGE	123	104	107	H42	
Zambia	SGE	123	104	107	H42	
Zambia	SGE	131	104	107	H66	SGE1
Zambia	SGE	131	104	107	H66	SGE1
Zambia	SGE	131	104	107	H66	SGE1
Zambia	SGE	131	104	107	H66	SGE1
Zambia	SGE	131	104	107	H66	SGE1
Zambia	SGE	131	104	107	H66	SGE1
Zambia	SGE	131	104	107	H66	SGE1
Zambia	SGE	131	104	107	H66	SGE1
Zambia	SGE	131	104	107	H66	SGE1
Zambia	SGE	131	104	107	H66	SGE1
Zambia	SGE	131	104	107	H66	SGE1
Zambia	SGE	131	104	107	H66	SGE1
Zambia	SGE	131	104	107	H66	SGE1
Zambia	SGE	131	104	107	H66	SGE1
Zambia	SGE	131	104	107	H66	SGE1
Zambia	SGE	131	104	107	H66	SGE1
Zambia	SGE	131	104	107	H66	SGE1
Zambia	SGE	131	104	107	H66	SGE1
Zambia	SGE	131	104	107	H66	SGE1
Zambia	SGE	131	104	107	H66	SGE1
Zambia	SGE	131	104	107	H66	SGE1
Zambia	SGE	131	104	107	H66	SGE1
Zambia	SGE	131	104	107	H66	SGE1
Zambia	SGE	131	104	107	H66	SGE1
Zambia	SGE	131	104	107	H66	SGE1
Zambia	SGE	131	104	107	H66	SGE1
Zambia	SGE	131	104	107	H66	SGE1
Zambia	SGE	131	104	107	H66	SGE1
Zambia	SGE	131	104	107	H66	SGE1
Zambia	SGE	131	104	107	H66	SGE1
Zambia	SGE	131	104	107	H66	SGE1
Zambia	SGE	131	104	107	H66	SGE1

Population ID	DHPS	0.8kb	4.3kb	7.7kb	2 locus haplotype code	Lineage code
Zambia	SGE	131	104	107	H66	SGE1
Zambia	SGE	131	104	107	H66	SGE1
Zambia	SGE	131	104	107	H66	SGE1
Zambia	SGE	131	104	107	H66	SGE1
Zambia	SGE	131	104	107	H66	SGE1
Zambia	SGE	131	104	107	H66	SGE1
Zambia	SGE	131	104	107	H66	SGE1
Zambia	SGE	131	104	107	H66	SGE1
Zambia	SGE	131	104	107	H66	SGE1
Zambia	SGE	131	104	107	H66	SGE1
Zambia	SGE	131	104	107	H66	SGE1
Zambia	SGE	131	104	115	H66	SGE1
Zambia	SGE	131	104	119	H66	SGE1
Zambia	SGE	131	104	119	H66	SGE1
Zambia	SGE	131	104	119	H66	SGE1
Zambia	SGE	131	104	121	H66	SGE1
Zambia	SGE	131	104	125	H66	SGE1
Zambia	SGE	131	104	134	H66	SGE1
Zambia	SGE	131	104	-	H66	SGE1
Zambia	SGE	131	104	-	H66	SGE1
Zambia	SGE	131	104	-	H66	SGE1
Zambia	SGE	131	104	-	H66	SGE1
Zambia	SGE	131	106	111	H67	SGE1
Zambia	SGE	131	106	-	H67	SGE1
Zambia	SGE	131	110	113	H69	SGE1
Zambia	SGE	131	110	125	H69	SGE1
Zambia	SGE	131	116	119	H72	
Zambia	SGE	131	-	-		
Zambia	SGE	133	104	107	H76	
Zambia	SGE	133	104	107	H76	
Zambia	SGE	133	104	117	H76	
Zambia	SGE	133	110	117	H79	
Zambia	SGE	-	104	107		
Zambia	SGE	-	104	113		
Zambia	SGE	-	104	-		
Zambia	SGE	-	104	-		
Zambia	SGE	-	104	-		
Zambia	SGE	-	104	-		
Zambia	SGK	117	106	141	H22	AGK/SGK1
Zambia	SGK	131	104	107	H66	
K1	SGK	131	104	107	H66	
3D7(A)	SGK	115	116	129		
