# Supplementary material for: Multiple Origins and Regional Dispersal of Resistant dhps in African Plasmodium falciparum Malaria
Source: PLoS Med. 2009 Apr 14;6(4):e1000055. doi: 10.1371/journal.pmed.1000055 (PMC2661256; doi:10.1371/journal.pmed.1000055)
Supplement: Text S1 — Details of samples used in this study, including informed consent and ethical approval. (0.11 MB DOC) [file pmed.1000055.s003.doc]

**Text S1**

**Sample details**

*Drug Abbreviations*

AQ, amodiaquine

SP, sulfadoxine-pyrimethamine

AQ-SP, amodiaquine+ sulfadoxine-pyrimethamine

AS+SP, artesunate+ sulfadoxine-pyrimethamine

AS+AQ, artesunate+amodiaquine

AL, artemether + lumefantrine (Coartem)

CD, chlorproguanil-dapsone (Lapdap)

CQ, chloroquine

SP+1AS, one dose of SP plus one dose of AS.

SP+3AS, one dose of SP plus AS given for three days.

SP+3AQ, one dose of SP plus AS given for three days.

3AQ+3AS, AQ plus AS both given for three days.

**Angola 2004**

***UigeTown, Uige Province***

Samples were collected from 73 children aged <10 years with uncomplicated malaria, who were seeking treatment at Uige Provincial Hospital, Uige Town during July-August 2004. Fingerprick bloodspots were collected for the genetic evaluation of parasite drug resistance. They were taken prior to treatment with the informed consent from their parent or guardian obtained by written procedures according to WHO protocol. [1]. Written informed consent was taken where literacy allowed, otherwise verbal consent was recorded by the clinicial and principle nurse who signed the following statement in the consent form ‘ We have explained the study to the households of – and are satisfied that he/she understands and consents’. Ethical approval to conduct the study was obtained from the Ministry of Health of Angola and the ethics committee of the London School of Hygiene and Tropical Medicine.

**Burkina Faso 2002**

***Ziniaré and Boussé health districts located north and northwest of Ouagadougou.***

Samples were collected from children aged 6 to 59 months with uncomplicated malaria, who were seeking treatment at health facilities in Ziniaré and Boussé during August - November 2002. Fingerprick bloodspots were collected for the genetic evaluation of parasite drug resistance as part of an *in vivo* study of CQ efficacy. They were taken before treatment after obtaining written, informed consent from their parents or guardian. Ethical approval to conduct the study was obtained from the Ministry of Health of Burkina Faso and the ethics committee of the London School of Hygiene and Tropical Medicine. Further details are published [2].

**Cameroon 2004**

***Limbe/Mutengene, Yaounde and Garoua***

Samples were collected and genotyped as part of an *in vivo* efficacy study in children aged 5-59 months during 2004 comparing AQ, SP and AQ-SP. Outpatients with uncomplicated malaria attending units of the Cameroon Baptist Convention Health Board clinics (Mutengene) and Hôpital Jesus, (Yaounde) Sauve et Guerit (Garoua) were enroled after written informed consent was obtained from parents/guardians and fingerprick bloodspots taken prior to treatment. Ethical approval was obtained from the Institution Review Board of the Cameroon Baptist Convention (CBC), the National Ethics Committee of Cameroon and from the ethics review committee of the London School of Hygiene and Tropical Medicine. Further details will be published (Mbacham et al in prep)

**Congo 2004**

***Kindamba , Kindamba district, Pool Province.***

Samples were collected and genotyped as part of a comparative trial on the efficacy of three artemisinin-based combinations (AS+SP, AS+AQ or AL). Children of 6-59 months attending the Kindamba Centre de Sante Integre Health Centre with uncomplicated malaria during May to October 2004 were enroled to the study after written informed consent was given by their parent or guardian and fingerprick samples taken prior to treatment. The protocol received ethical clearance from the Ministry of Health of the Republic of Congo and the external Ethical Review Board used by Médecins sans Frontières. The necessary permission from the local authorities (Sous-prefet and hospital senior staff) in Kindamba, Pool District of the study was obtained. Further details are published [3]

**Democratic Republic of Congo 2004**

***Shabunda, South Kivu Province.***

Samples were collected during April-May 2004 from children aged between 6-59 months attending the ‘Divine Maitre’ Health Centre of Shabunda with uncomplicated *P.falciparum* malaria. The genotyping of pre-treatment samples was carried out as part of an evaluation of in-vivo efficacy of AS+AQ and AS+SP. Parents or guardians of children gave informed written consent before inclusion of their child in the study. The ethics committee of the DRC National Malaria Programme and the external Ethics Review Board used by Médecins sans Frontières reviewed and approved the study protocol. Further details are published [4]

**Ethiopia 2004**

***Humera, Ethiopia***

Samples were collected from patients of all ages who attended Kahsay Abera Hospital in Humera during January-April 2004 with symptomatic uncomplicated malaria. Patients were enroled an in-vivo efficacy trial, comparing AL with SP which was conducted by staff of Kahsay Abera Hospital in Humera and the Mekele Regional Health Bureau. Fingerprick bloodspot samples were taken from patients prior to treatment after written informed consent for participation in the study was given, and genetic analysis carried out in support of the drug efficacy evaluation. Ethical permissions for the study were given by the Ethical Clearance Committee of the Tigray Health Research Council and the external Ethics Review Board used by Médecins sans Frontières.

**Gabon 2007**

***Lambaréné***

Pre-treatment samples were collected from children between 6-59 months with uncomplicated *P.falciparum*  malaria confirmed by blood film. The genotyping was carried out as part of an evaluation of in-vivo efficacy of SP during 2007. Parents or guardians of children gave written informed consent for inclusion of their child in the study unless this was impossible to obtain in case of illiteracy, in which case witnessed oral consent was obtained and documented in writing. Ethical permissions were given by the ethics committee of the Comite d’Ethique Regional Independent de Lambaréné (CERIL), the internal ethics committee of the Albert Schweitzer Hospital, Lambaréné, Gabon and London School of Hygiene and Tropical Medicine ethics committee. Further details are published in [5]

**The Gambia 2004**

***Farafenni and Njaba Kunda,***

Samples were from children aged 6 months to 10 years attending Farafenni Maternal and Child Health Clinic in Farafenni and Njaba Kunda, a mission clinic in a rural village. Children with uncomplicated malaria were enroled in a randomized trial of safety and effectiveness of CD (Lapdap) compared to AL during Oct-Dec 2004. Samples taken prior to treatment after the child’s parent or guardian had given written informed consent. Ethical approval to conduct the study was obtained from the joint ethics committee of Gambia Government and MRC Laboratories. The Trials database entry: [www.clinicaltrials.gov](http://www.clinicaltrials.gov/) identifier NCT00118794. Further details will be published in Dunyo et al in prep.

**Ghana 2003**

***Navrongo, Kassena-Nankana District***

Samples were taken from asymptomatic children aged **19-41 months (average age 30 months**) who were screened during November 2003. Finger prick blood sample for blood slide and filter paper blood spots were collected from each child, and parasite positive individuals were enroled into an open label SP efficacy study. Molecular analysis was carried out as part of the drug efficacy evaluation. Parents or guardians of children gave informed written consent before inclusion of their child in the study. Ethical approval to conduct the study was obtained from the Ministry of Health of Ghana and the ethics committee of the London School of Hygiene and Tropical Medicine. Further details will be published in Chandramohan et al in prep.

**Ghana 2005**

***Hohoe***

A cross sectional survey was carried out of children aged 3-59 months who were participating in an IPTc study **comparing SP,** AS+AQ **or placebo** during December 2005, one year after the end of the intervention. They were invited to a central point in the community and finger prick blood sample for blood slide and filter paper blood spots were collected from each child. Most of parasite positive children were asymptomatic at the time of the survey. Molecular analysis was carried out as part of the drug efficacy evaluation. Parents or guardians of children gave informed written consent before inclusion of their child in the study. Ethical approval to conduct the study was obtained from the Ministry of Health of Ghana and the ethics committee of the London School of Hygiene and Tropical Medicine. The Trials database entry: [www.clinicaltrials.gov](http://www.clinicaltrials.gov/) identifier NCT00119132. Further details will be published (Kweku et al in prep)

**Guinea 2004-2005**

***Lainé refugee camp, Lola prefecture , Republic of Guinea.***

Patients attending the Médecins sans Frontières health centre in Lainé refugee camp during December 2004 - January 2005 with confirmed *Plasmodium falciparum* malaria had a blood sample collected for genotypic analysis as part of an in-country evaluation of drug efficacy. The camp has 22,000 Liberian refugees, is located in Lola prefecture, N’Zerekore region which borders Ivory Cost and Liberia. Médecins sans Frontières has been present since 2002 and runs the only health centre of the camp. Written informed consent was obtained from each included patient or their carer. Ethical approval to conduct the study was given by the Ministry of Health of the Republic of Guinea. Further details are published [6]

**Kenya 2006**

***Bondo District, Western Kenya.***

Samples were collected from schoolchildren aged 5-18 years taking part in an evaluation of intermittent preventive treatment during March 2006. After the study aims and procedures were explained in the local language, individual written consent was obtained from the parents/guardians and witnessed by a second party. Asymptomatic *P. falciparum* infections were identified during a cross-sectional survey of 30 schools in Bondo District and they were genotyped as part of the post-intervention evaluation of intermittent preventive treatment with SP+AQ. Ethical approval for the study was given by the Kenyatta National Hospital Ethical Review Committee and the ethics committee of the London School of Hygiene and Tropical Medicine. The Trials database entry: [www.clinicaltrials.gov](http://www.clinicaltrials.gov/) identifier NCT00142246. Further details are published [7].

**Mozambique 2001**

***Maputo Province.***

Samples were systematically collected in parasite prevalence surveys in a random community-based sample of children aged 2 - 14 years at sentinel sites in Maputo Province, southern Mozambique. Fingerprick samples were taken after informed verbal consent from parent or guardian was given. Verbal, rather than written, consent was sought because of high illiteracy rates (>40%). Surveys and molecular analysis were conducted as part of a programme of regional surveillance of drug efficacy within the Lubombo Spatial Development Initiative (LSDI). The LSDI is a cross-border collaboration between the governments of Mozambique, Swaziland and South Africa. Ethical approval for the study was obtained from the South African Medical Research Council and the Maputo Province Directorate of Health, Mozambique. Further details are published [8,9].

**Namibia 2005**

***Kavango Region***

Samples were collected from patients of all ages attending health facilities in Andara Health District (Andara Hospital, and Divundu, Old Bagani , Omega and Shadikongoro Clinics) and in Rundu Health District (Kaisosi, Ndama, Nkarapamwe, Rundu, and Sauyemwa Clinics) during April and May 2005. These patients had uncomplicated *Plasmodium falciparum* malaria confirmed by rapid diagnostic test. Genotypic analysis of parasites was carried out with the written informed consent of patients as part of an in-country evaluation of drug efficacy. Ethical approval to conduct the study was obtained from the Namibian Ministry of Health and Social Services and the ethics committee of the London School of Hygiene and Tropical Medicine.

**Nigeria 2005**

***Abuja***

Samples were collected from patients of all ages who were attending the National Hospital Abuja with cases of uncomplicated *Plasmodium falciparum* malaria confirmed by rapid diagnostic test. Each patient donated a blood sample for genotypic analysis as part of an in-country evaluation of drug efficacy after giving their written informed consent. Ethical approval to conduct the study was obtained from the *National Hospital Abuja* ethical committee and the ethics committee of the London School of Hygiene and Tropical Medicine.

**Senegal 2004**

***Niakhar, Central Senegal***

Asymptomatic infections among children aged 2-59 months were identified and genotyped as part of a study comparing 4 treatment regimens (SP+1AS, SP+3AS, SP+3AQ, and 3SP+3AQ) for seasonal intermittent preventive treatment for prevention of malaria in children. The study was done in Niakhar a rural district in central Senegal and the parents/guardians of all children participating gave written informed consent. Finger-prick samples were collected for parasitological screening and genotyping prior to being given the first dose of treatment during September 2004. Ethical approval for the study was obtained from the ethics review boards of the Senegalese Ministry of Health and London School of Hygiene and Tropical Medicine. An independent data safety monitoring board monitored the trial. The Trials database entry: [www.clinicaltrials.gov](http://www.clinicaltrials.gov/) identifier NCT00132561. Further details are published [10].

**Sudan 2003**

***Daraweesh and Kajara (15km from Gedaref in eastern Sudan)***

The samples were collected from patients of all ages with uncomplicated malaria attending a clinic which serves the villages of Daraweesh and Kajara (15km from Gedaref in eastern Sudan) during October-December 2003. Patients were enrolled according to WHO 2003 recommendations, and were treated either with SP+CQ or with SP monotherapy. Individual informed verbal consent of all patients, and the parents/guardians of all children participating was a requirement for inclusion in the study. Verbal, rather than written, consent was sought so as not to interrupt the routine diagnosis and treatment of patients at the health facility during the malaria season. The community has participated in a longitudinal study of malaria for 20 years and verbal consent is the accepted form of consent agreed by the inhabitants and the village leaders. Finger-prick samples were collected for parasitological screening and genotyping prior to treatment Ethical Approval for the study was given by the Ministry of Health, Sudan. Further details are published in [11,12]

**Tanzania 2004**

***Kilombero Ulanga Districts***

Samples were collected from children and adults whose infections were identified in cross sectional community surveys in the rural districts of Kilombero and Ulanga conducted during July- Sept of 2004. The surveys were part of large combination therapy pilot implementation programme in Tanzania, the Interdisciplinary Monitoring Programme for Antimalarial Combination Therapy (IMPACT-TZ). A finger-prick blood sample for blood slide and filter paper bloodspot were collected from each individual for molecular genotyping. Written informed consent was obtained from all individuals or their guardians before collection of samples. Scientific and ethical clearance was granted from the Medical Research Council of the National Institute for Medical Research in Tanzania, the Centers for Disease Control and Prevention, USA, and the London School of Hygiene and Tropical Medicine. IMPACT-Tanzania is a multiyear implementation research evaluation that rests on a collaborative platform incorporating the US Centers for Disease Control and Prevention (CDC), the Ifakara Health Research and Development Centre, London School of Hygiene and Tropical Medicine, and the Ministry of Health and Social Welfare including its National Malaria Control Programme, the Tanzania Essential Health Interventions Project and the Council Health Management Teams of Rufiji, Kilombero, Ulanga, Morogoro and Mvomero Districts. IMPACT-Tanzania is primarily supported by funding from the United States Agency for International Development, CDC and Wellcome Trust.

**Uganda 2005**

***Rukungiri district and Kabale districts in Southwest Uganda.***

Fingerprick bloodspots were from symptomatic malaria patients of all ages presenting at Kebisoni Health Facility (Rukungiri), Bufundi Health Facility (Kabale) and clinical diagnosis confirmed using rapid diagnostic tests. Verbal informed consent was sought from all patients or their guardians before the collection of fingerprick bloodspot samples using a study information sheet with standard format. Consent was noted by the clinician for each individual patient in a section of the sheet for recording informed consent. Verbal (rather than written) consent was sought so as not to interrupt the normal flow of patients at the health facility and to avoid increased workload for the health facility staff. Scientific and ethical permissions given by the Uganda National Council for Science and Technology (UNSCT HS 35) and the ethics committee of the London School of Hygiene and Tropical Medicine. Further details are published in [13].

**Zambia 2005**

***Mpongwe, Chipata, Mansa, Isoka, Chongwe, and Chibombo.***

Fingerprick bloodspots were from symptomatic malaria patients of all ages presenting at health facilities in Mpongwe (Copperbelt Province), Chipata, (Eastern Province), Mansa (Luapula Province), Isoka, (Northern Province), Chongwe (Lusaka Province), Chibombo (Central Province) during Jan-April 2005. Clinical diagnosis was confirmed with Giemsa-stained thick films and rapid diagnostic tests. Samples were taken prior to treatment and written informed consent was obtained from all individuals or their guardians before the collection of samples. Molecular analysis was carried out as part of an in-country evaluation of drug efficacy. Scientific and ethical permissions were given by the National Ministry of Health and the ethics committee of the London School of Hygiene and Tropical Medicine. Further details are published in [14]
