# Supplementary material for: Multiple Origins and Regional Dispersal of Resistant dhps in African Plasmodium falciparum Malaria
Source: PLoS Med. 2009 Apr 14;6(4):e1000055. doi: 10.1371/journal.pmed.1000055 (PMC2661256; doi:10.1371/journal.pmed.1000055)
Supplement: Text S2 — Literature search strategy and terms. (0.03 MB DOC) [file pmed.1000055.s004.doc]

**Text S2**

**Literature search strategy:**

A literature search was done using the National Library of Medicine search engines, Pubmed and Medline. We selected articles published in English and search terms included Africa, *dhfr* , *dhps* sulphadoxine, sulfadoxine, pyrimethamine, Fansidar, Africa, prevalence, malaria and resistance as free text. Studies were not restricted to randomized controlled trials. We read each abstract and assessed whether or not the article was suitable. Hardcopies of papers matching the criteria were obtained and read fully. The *dhps* data forms a sub-set of this. Inclusion and exclusion criteria for selecting published studies specifically on *dhps* 436,437 and 540 haplotypes are listed in Table 1. We found 20 suitable studies that reported data on *dhps* codons 436, 437 and 540 in the required time frame. Where there was more than one study for a geographical region, the most recent was taken. Since the search one of own studies has been published and the citation for this now appears, increasing the number of published studies to 21.

Table 1: Search terms, Inclusion and Exclusion criteria for selecting *dhps* studies on codons 436,437 and 540

|  | Inclusion | Exclusion |
| --- | --- | --- |
| Parasite species | *P. falciparum* | All others |
| Time of study | 1997 to present | pre-1997 |
| Language | English | Non-english |
| Gene | *dhps* or *dhps* and *dhfr* | Papers reporting *dhfr* alone |
| Point mutation haplotype | Combined *dhps* haplotype of point mutations at codons 436 437 and 540 | *dhps* point mutation data but haplotypes not reported. Haplotypes which did not include all three codons (436 437 and 540). |
| Search terms | *dhfr dhps* sulphadoxine, sulfadoxine pyrimethamine Fansidar Africa prevalence malaria resistance |  |
